# *Drosophila melanogaster* grooming possesses syntax with distinct rules at different temporal scales

**DOI:** 10.1371/journal.pcbi.1007105

**Published:** 2019-06-26

**Authors:** Joshua M. Mueller, Primoz Ravbar, Julie H. Simpson, Jean M. Carlson

**Affiliations:** 1 Interdepartmental Graduate Program in Dynamical Neuroscience, University of California, Santa Barbara, Santa Barbara, California, United States of America; 2 Department of Physics, University of California, Santa Barbara, Santa Barbara, California, United States of America; 3 Department of Molecular, Cellular, and Developmental Biology, University of California, Santa Barbara, Santa Barbara, California, United States of America; Princeton University, UNITED STATES

## Abstract

Mathematical modeling of behavioral sequences yields insight into the rules and mechanisms underlying sequence generation. Grooming in *Drosophila melanogaster* is characterized by repeated execution of distinct, stereotyped actions in variable order. Experiments demonstrate that, following stimulation by an irritant, grooming progresses gradually from an early phase dominated by anterior cleaning to a later phase with increased walking and posterior cleaning. We also observe that, at an intermediate temporal scale, there is a strong relationship between the amount of time spent performing body-directed grooming actions and leg-directed actions. We then develop a series of data-driven Markov models that isolate and identify the behavioral features governing transitions between individual grooming bouts. We identify action order as the primary driver of probabilistic, but non-random, syntax structure, as has previously been identified. Subsequent models incorporate grooming bout duration, which also contributes significantly to sequence structure. Our results show that, surprisingly, the syntactic rules underlying probabilistic grooming transitions possess action duration-dependent structure, suggesting that sensory input-independent mechanisms guide grooming behavior at short time scales. Finally, the inclusion of a simple rule that modifies grooming transition probabilities over time yields a generative model that recapitulates the key features of observed grooming sequences at several time scales. These discoveries suggest that sensory input guides action selection by modulating internally generated dynamics. Additionally, the discovery of these principles governing grooming in *D. melanogaster* demonstrates the utility of incorporating temporal information when characterizing the syntax of behavioral sequences.

## Author summary

Analysis of temporally rich behavioral sequences provides a quantitative description of the rules underlying their generation. *Drosophila melanogaster* grooming behavior consists of many complex sequences involving repetitions of well-characterized actions. In this paper, we leverage advances in machine vision to automatically annotate over 40 hours of video data of flies covered in dust and develop mathematical models that reveal the existence of syntax in *D. melanogaster* grooming. We find that sequence organization depends on grooming action identity, as has been well-established, and, more surprisingly, grooming action duration. The discovery of duration-dependent action selection leads us to conclude that, although sensory input informs grooming decisions on long time scales, internal dynamics also guide individual transitions between grooming actions. Therefore, incorporating action duration into our models allows us to uncover multi-scale temporal dynamics that suggest the existence of neural circuits dedicated to partially sensory-independent decision-making. Our approach highlights the importance of incorporating temporal information into sequential models, as doing so reveals the relative contributions of sensory input and internal dynamics to behavioral sequence generation.

## Introduction

Sequential animal behaviors are often composed of repetitions of simple subroutines. In driving motor action execution, nervous systems must integrate sensory information with internal priorities and dynamics. Both external and internal conditions contribute to sequence organization, but their respective weights are unknown. At one extreme, purely sensory-driven, reflexive processes produce actions, such as larval escape sequences, solely based on recent sensory input [1]. At the other, internally generated and maintained nervous system dynamics, such as those found in the crustacean stomatogastric ganglion, may produce behaviors which proceed irrespective of changing external conditions [2, 3].

Grooming, a common behavior across species, confers social and survival benefits [4, 5] and provides a rich source of data for discovering rules that organisms use to produce behavioral sequences. The vocabulary of grooming consists of the possible actions that may be executed and has been cataloged in mice and several species of flies [6–8]. However, the rules for grooming action sequence organization, or syntax, remain poorly understood. In *Drosophila melanogaster*, or fruit flies, these sequences are variable between individuals and within individuals across grooming sessions, suggesting that flies use non-deterministic rules to make grooming decisions. Because of the complex structure of these sequences, analysis of *D. melanogaster* grooming can reveal distinct rules of sequence generation at different temporal scales: the long time scale of bulk behavioral progression, the intermediate scale of grooming motifs, and the short scale of individual grooming bouts.

On long time scales, *D. melanogaster* grooming in response to exposure to an irritant is well-described as a process that typically progresses from an early phase characterized by preferential grooming of anterior body parts, such as eyes, to a later phase which exhibits heightened proportions of walking, abdomen cleaning, and wing cleaning. This progression occurs gradually over the course of many minutes. Previously published computational models which utilize either hierarchically structured suppression or graded sensory gain exhibit gross features of this progression, namely a gradual transition from early anterior-heavy grooming to a later quasi-steady state featuring increased posterior grooming and walking levels [9].

At an intermediate temporal scale, grooming is organized in units that we refer to as motifs. We identify two classes of motifs (anterior and posterior), which are named for the set of legs used to execute them (front and back, respectively). Motifs consist of consecutive alternations between body-directed grooming bouts and leg rubbing bouts. Bouts are defined as sustained periods of a single grooming action (e.g. head cleaning or wing cleaning). Grooming bouts occur at the shortest time scale we consider here, as individual bouts typically last somewhere between 150 ms and 2 s. Flies use the same pair of legs to execute bouts within a motif, allowing them to transition between within-motif actions easily. Specifically, an anterior grooming motif consists of consecutive alternations between bouts of head cleaning and front leg rubbing. A posterior motif consists of alternating bouts of abdomen cleaning, wing cleaning, and back leg rubbing.

Analysis of freely-behaving flies reveals behavioral structure at multiple time scales, with coarse-grained descriptions being sufficient to describe long-term trends and higher resolution descriptions providing increased predictive power at shorter time scales [10]. This finding suggests that there is value in examining sequential data at several levels of temporal resolution. Additionally, it suggests that treating behaviors as both continuously varying and discretely separated in the same analysis can yield more insight than considering either purely continuous or discrete models. Currently, we do not know how flies integrate sensory information with internal states in order to produce multi-scale grooming behavior. It is possible that long time scale grooming trends are governed principally by changing sensory conditions, as grooming results in the removal of irritant over time. Several studies also provide evidence that sensory input is sufficient to drive grooming behavior in a reflex-like fashion at short time scales [11, 12]. Here, we use statistical models to characterize grooming syntax at each of the temporal scales mentioned above.

Several classes of Markov models, in which the probability of an event occurring is contingent upon previous events, have been used to describe factors involved in non-deterministic decision-making in various animals. Different Markov models vary in their structure and number of parameters but they each require a well-defined state space. Navigation and foraging have been described using Markov models, as these sequential behaviors can be decomposed into subroutines which can be categorized using easily observable dimensions such as direction and velocity [13–15]. These models are applicable to common behaviors exhibited by *D. melanogaster* as well, such as courtship and locomotion [16–18]. Recent work from Tao et al. uses a Hierarchical Hidden Markov Model (HHMM) to analyze fruit fly locomotor behavior in the presence of odors [19]. They find that, although locomotor behavior is non-stereotyped, it can be decomposed into stereotyped units, making it suitable for analysis within a Markovian framework. Berman et al. [20] use high-order Markov models to describe the behavior of freely-roaming *D. melanogaster*, suggesting the applicability of such models to behavioral subsets identified in their analysis, including grooming behavior.

It should be noted that non-Markovian dynamics have also been identified in animal vocal sequence production, suggesting that other classes of models may be useful for describing behavioral generation [21–23]. In fact, Berman et al. [20] report that, although a Markovian framework is useful for illustrating some features of behavioral transitions, they observe long time dependencies in their data that cannot be captured by their Markov models.

**Fig 1:**
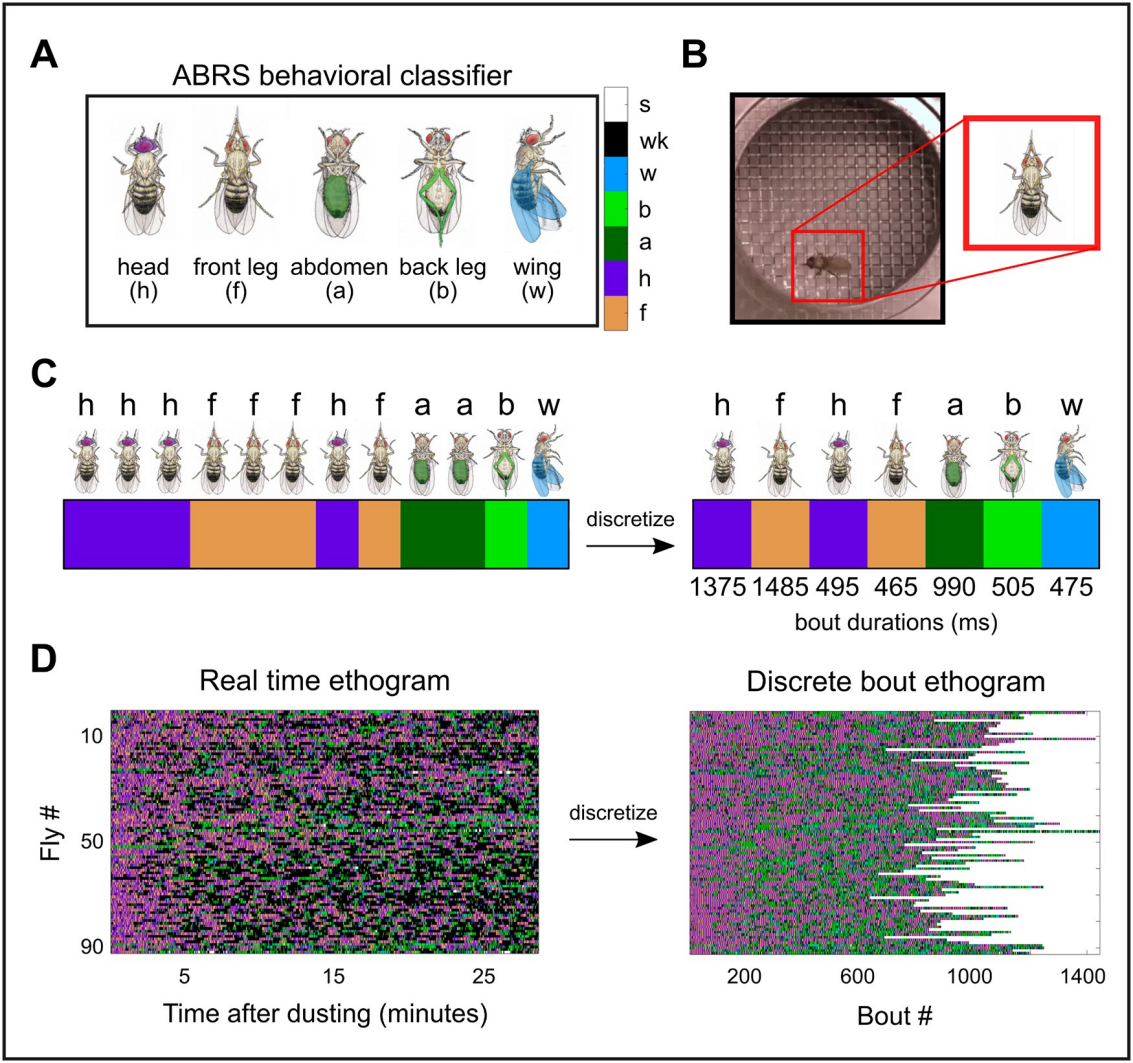
Applying the ABRS classifier to video data generates large-scale ethogram data sets. A: The ABRS classifier is trained to annotate video data with labels corresponding to one of five grooming actions observed in *D. melanogaster* (front leg rubbing = f, head cleaning = h, abdomen cleaning = a, back leg rubbing = b, wing cleaning = w) or two non-grooming actions (walking = wk, standing = s, not shown). Color bar (right) displays the corresponding color code used for visualization of grooming action sequences. B: Flies are covered with dust, placed in a chamber, and recorded from above. Video data is then passed through the ABRS classifier, which locates the fly in each frame and applies a behavioral label. Here, the fly is shown performing a front leg rub. C: After classification, video data can be represented by a vector. Each vector element represents one frame and contains a behavioral label, visualized here using a color code. Without loss of information, the real time ethogram (left) is recast as a discrete bout ethogram, defined by discrete transitions between individual bouts, which are labeled by both action and duration. We refer to this process as “discretizing” the ethogram. This results in a vector (right) containing both an ordered list of grooming action labels (color) and grooming action durations (number). For example, the first bout shown here consisting of consecutive frames of head grooming would be discretized such that its corresponding action label would be “h”, indicated by the purple block. For illustrative purposes, we choose bout durations, shown below the discrete bout ethogram, that are representative of the observed distributions. We refer to instances of sustained identical grooming actions as bouts. D: Real time ethograms for 92 dusted flies (left) are shown as a matrix containing 92 rows and 50,000 columns. After discretization, ethograms contain variable numbers of bouts but retain information about bout order and bout duration (right).

Here, we use our in-house Automatic Behavior Recognition System (ABRS, https://github.com/AutomaticBehaviorRecognitionSystem/ABRS/), which can classify different grooming movements from videos of flies covered in dust, to generate a large data set of ethograms—records of cleaning actions over time—from wild-type flies removing dust. We analyze more than 40 total hours of video from over 90 flies (Fig 1).

**Fig 2:**
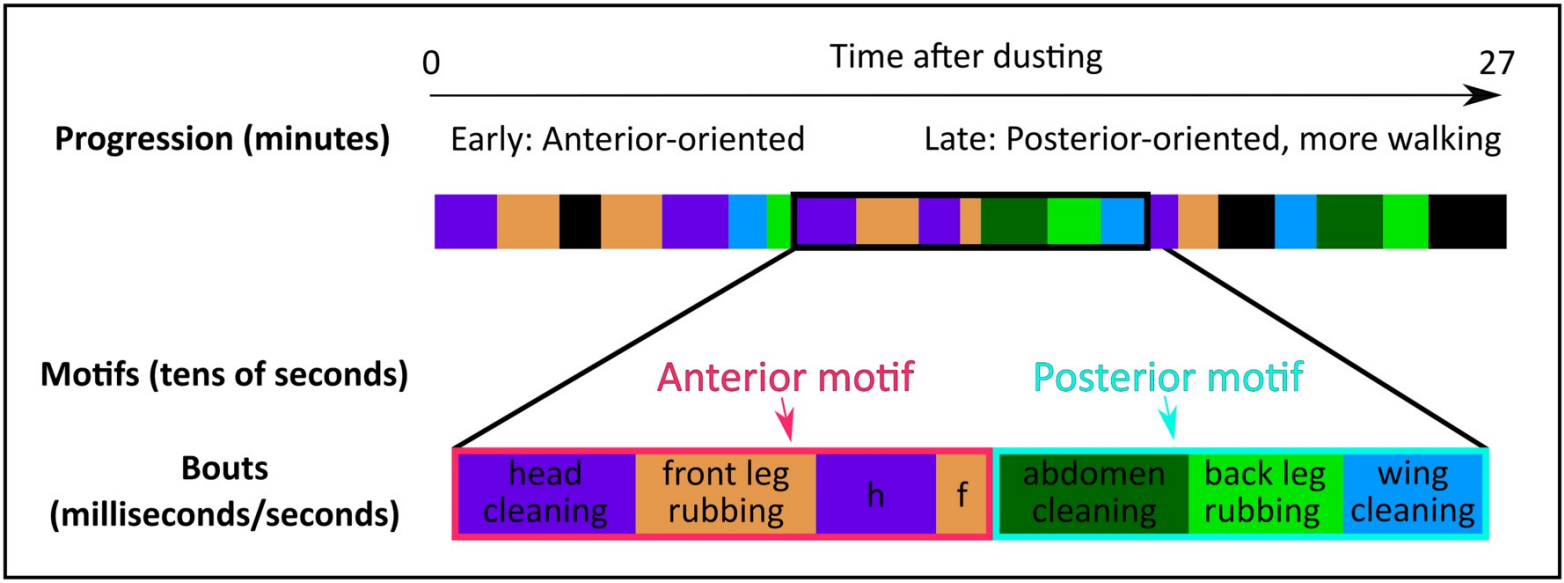
Schematic overview of the multiple time scales present in *Drosophila melanogaster* grooming ethograms. Grooming ethograms possess at least three distinct time scales. The grooming progression (top), which takes many minutes to observe in its entirety, occurs at the longest time scale we consider here. We refer to this phenomenon as a progression because we observe that, on average, flies begin grooming with anterior-oriented actions and end with posterior-oriented actions and more walking. At an intermediate time scale, ethograms consist of grooming motifs (middle). Motifs are composed of consecutive grooming bouts which use the same pair of legs (either anterior or posterior). At the shortest time scale, we observe individual grooming bouts (bottom), which consist of sustained grooming movements that range from several hundreds of milliseconds to a few seconds in duration.

First, this data set allows us to quantify behavioral trends at several temporal scales on a larger data set than has been previously described. Fig 2 provides a schematic overview of the temporal scales we analyze here. Notably, we observe the progression from anterior to posterior grooming that has been identified by Seeds et al. [9]. We also observe a strong correlation between body-directed and leg-directed cleaning actions within motifs. That is, the amount of head cleaning and front leg rubbing are extremely strongly correlated over the entire course of grooming, as are the amount of back leg rubbing and the sum of abdomen and wing cleaning. Finally, we observe a relaxation into steady state-like behavior after approximately 13 minutes of grooming.

Next, we use a set of Markov models to characterize probabilistic rules governing grooming action transitions. We use a Markovian framework as a first approximation in order to discover relevant features of grooming, since Markov models are simple and carry few assumptions. As such, these models serve as a tool for grooming sequence feature identification rather than as a full explication of grooming decision-making factors. Previous work has indicated that such analysis can reveal temporal relationships in sequential behavioral data, indicating that, even if the behavior being analyzed is not fully Markovian in reality, Markov models can still be useful as a descriptive exploratory tool [16–20].

The simplest of our models, a first order discrete time Markov chain, highlights the presence of grooming motif structure. Subsequent models incorporate temporal information by considering grooming bout durations. By comparing these models to statistical null model hypotheses, which shuffle bout order or bout duration, we discover the contribution of bout duration to grooming sequence structure at short time scales.

Though we rely on a Markovian framework to identify features of grooming syntax, our analysis does not preclude the possibility of non-Markovian dynamics in fruit fly grooming. Indeed, our data exhibits two notable nonstationarities. First, we observe an overall trend in grooming proportions, as has been reported previously [9], wherein flies favor anterior grooming immediately after being dusted but favor posterior grooming and walking after approximately 10-15 minutes. Second, grooming transition probabilities change slightly over the course of grooming (though they are unexpectedly stationary over time), indicating that a purely stationary model is an oversimplification. To account for this, we extend our analysis to include a simple nonstationarity which captures sequence progression more closely than a first order Markov chain.

We find that a time-varying Markov renewal process (MRP) which incorporates bout duration dependence recapitulates the observed grooming structure at long, intermediate, and short time scales. This model is partially Markovian, as transition probability matrices dictate state transition dynamics. However, the model also includes renewal process dynamics, in which the duration of the ensuing bout is determined using a random draw from the empirically observed bout duration distribution. This model is nonstationary due to the fact that Markov transition probabilities change over time. This model recapitulates both the observed bulk grooming progression statistics and the fine-grain temporal structure of grooming motifs, suggesting that sensory drive can modulate internal programs in a simple manner to prioritize different grooming strategies in different contexts.

Our models clarify the syntax of grooming, demonstrating that, at the scale of individual bouts, transitions are dictated by both the identity and duration of the preceding bout. While previous studies have established the non-random nature of fruit fly grooming [6, 9, 16–18, 20], this work reveals the previously unknown contribution of grooming bout duration to sequence structure, suggesting a role for sensory input-independent decision-making. Finally, a model that includes duration dependence at the scale of individual grooming decisions and a linear progression to mimic dust removal is able to generate synthetic ethograms that closely resemble observed data at the intermediate scale of motifs and the long scale of the grooming progression.

The discovery of duration dependence in grooming sequence structure suggests the existence of neural circuits in *D. melanogaster* that can generate patterned behavior irrespective of sensory drive, as transition rules and bout durations remain nearly constant even as the dust distribution varies over the course of grooming. Additionally, the tight correlation between body and leg-directed actions within motifs demonstrates for the first time the existence of duration dependence at an intermediate temporal scale. Finally, the model improvements provided by the inclusion of a simple nonstationarity suggest that sensory drive plays a role in guiding the implementation of internal programs over long time scales rather than serving a purely reflexive action selection function at short time scales. These discoveries provide the groundwork for future experiments and analysis of the neural underpinnings of duration-dependent action selection and sensory-driven modulation of decision-making in grooming and related behaviors.

## Methods and models

### Ethogram generation

*Drosophila melanogaster* (N = 92) were recorded at 30 Hz for 27.8 minutes after being uniformly covered in Reactive Yellow 86 dust (Organic Dyestuffs Corporation, Concord, North Carolina). To generate labeled ethograms, we apply the ABRS classifier to video (https://github.com/AutomaticBehaviorRecognitionSystem/ABRS). Following this, real time ethograms are converted to discrete bout ethograms and de-noised (Fig 1).

Briefly, the classifier is trained on grooming data using a combination of supervised and unsupervised protocols in several steps. First, a Fourier transform is performed on pixel intensity data across 17-frame sliding windows (∼0.5 s), resulting in two-dimensional spectra referred to as space-time images (ST-images). ST-images are subsequently modified by Radon transformation, which can be used to represent 2D shapes in a rotation and translation-invariant manner. Fast Fourier transformation is then applied to the Radon transformed ST-images, resulting in position and orientation-invariant ST-images [24].

ST-images are then decomposed using singular value decomposition (SVD) and clustered using linear discrimination analysis (LDA) with human labeled behavioral categories. Clusters identified using LDA correspond to five predefined grooming behaviors (f = front leg rubbing, h = head cleaning, a = abdomen cleaning, b = back leg rubbing, w = wing cleaning) and two non-grooming behaviors (wk = walking, s = standing). After classification, each video is represented by a 50,000-element vector, with each element containing a label denoting the behavior identified in the corresponding frame. Classifier performance and validation are shown in S1 Fig.

To generate discrete bout ethograms, each 50,000-element vector is transformed into a two-dimensional vector in a series of steps. The first row of this discrete bout ethogram contains the bout identity, or the label corresponding to one of the seven behaviors described above. The second row contains the bout durations, or the amount of time spent in the corresponding behavior before transitioning to another behavior.

Consecutive frames containing identical actions are consolidated into one bout identity entry. Then, the corresponding bout duration entry is calculated by counting the number of consecutive identical frames and converted into seconds by dividing by the video frame rate (30 Hz). As a result, the number of entries in discrete bout ethograms corresponds to the number of observed bouts, which varies between flies. However, the sum of bout durations for each ethogram is identical (27.8 minutes).

After ethogram generation, behavioral bouts persisting for less than 167 ms are deemed artifacts and deleted. This threshold is chosen because manual inspection of video data suggests that individual leg sweeps occur at approximately this time scale. This is also the approximate average transition time between behavioral states described by Berman et al. [10]. Deleted bouts account for 1.03% of total recording time. In subsequent analysis, we also neglect standing bouts, as they account for < 1% of total recording time.

### Data binning

In order to include bout duration as a data dimension, we introduce a binning scheme. To implement this change, the continuous-valued entries in the second row of discrete bout ethograms are collapsed into duration category labels. Grooming bouts are classified into three categories (short, medium, and long) based on the duration of the associated bout. Although our results are consistent even when using more than three duration categories (S2 Fig), we limit analysis to three categories for the sake of simplicity.

**Fig 3:**
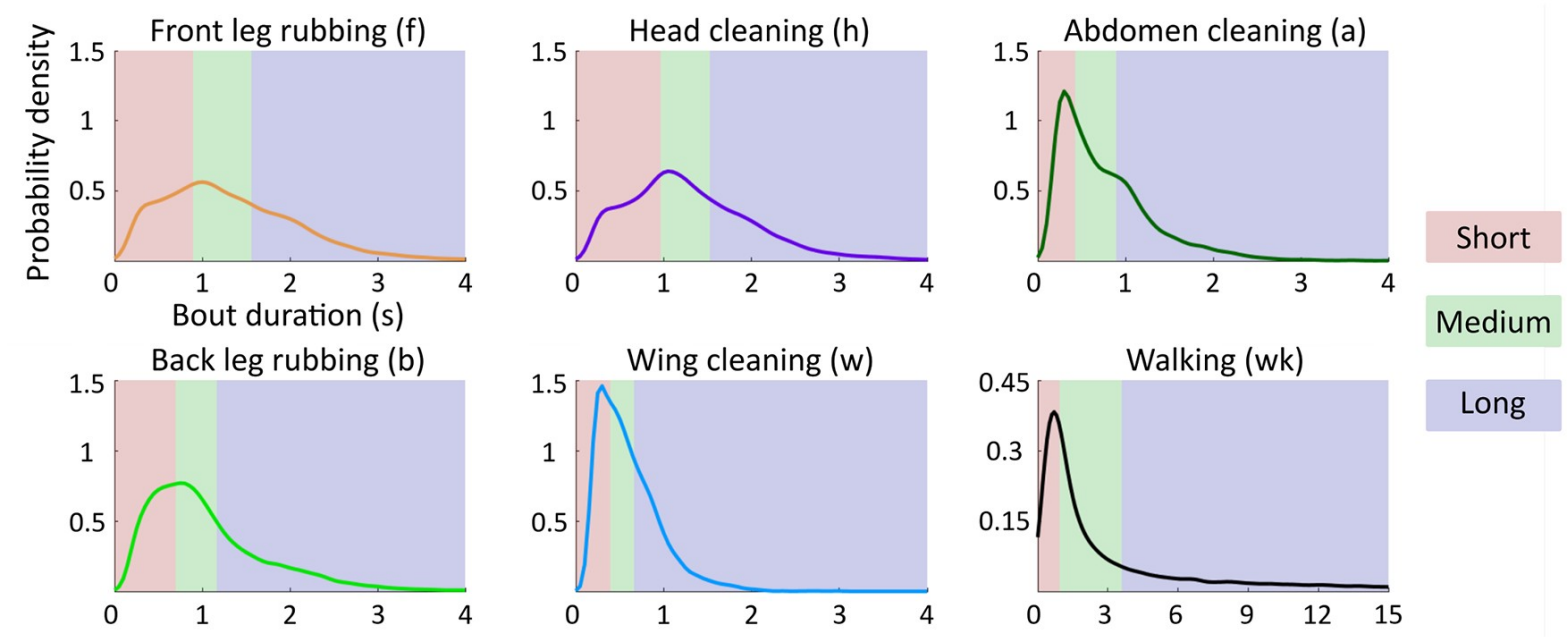
Binning grooming bouts by duration allows for analysis of temporal dependence. Ethograms are modified by introducing a binning scheme based on grooming bout duration. Here, we show the binning strategy used to create three duration categories for grooming actions. Grooming actions are classified as short, medium, or long bouts, indicated by the red, green, and blue regions of the probability density functions, respectively. Region boundaries are chosen such that each duration category contains an equivalent number of samples. Notably, anterior motif actions (f, h) possess strikingly similar duration distributions, suggesting coupling between these actions.

Bin edges for these categories are determined independently for each grooming action (f, h, a, b, w) and walking (wk), as each action possesses a unique duration distribution. Bin edges are chosen such that each bin is populated by an equal number of samples (i.e. the total number of actions labeled “short” is equal to the number of “long” actions) and are depicted in Fig 3.

### Markov chain analysis

Markov processes are a class of probabilistic models that describe transitions between states of a system in terms of previous states. The simplest version obeys the Markov property, which declares that the next transition between states depends only on the current state. As such, long-term history dependence can be neglected when considering Markov processes, greatly simplifying the models. In discrete time, the dynamics of a first order Markov chain are described by the equation

$$\begin{array}{c} x_{t+1}=x_{t}M, \end{array}$$ (1)

where **x**_*t*_ denotes the state probability vector at time *t*. The entries of **x**_*t*_ represent the probability of the system being in the state corresponding to that entry at time *t*. **M** is the matrix of state transition probabilities such that entry **M**_*ij*_ is the probability of transitioning from state *i* to state *j*. The vector **x**_*t*_ will have dimensionality corresponding to the number of possible states in the system. In this case, there are either six, twelve, or eighteen grooming states, depending on whether grooming bout duration is incorporated into the model. Given sufficient data about state transitions in a real system, it is possible to generate a maximum likelihood estimate (MLE) transition probability matrix, denoted by $\hat{M}$, which provides the best estimate of the transition probabilities governing the observed transitions. The entries of $\hat{M}$ are given by

$$\begin{array}{c} {\hat{M}}_{ij}=\frac{n_{ij}}{\sum_{u=1}^{v}n_{iu}}, \end{array}$$ (2)

where *n*_*ij*_ is the total number of observed transitions from state *i* to state *j*. The summation over *n*_*iu*_ is a normalization factor which counts the total number of transitions from state *i* to any other state, indexed by *u*, and *v* denotes the total number of possible states.

First order Markov chain maximum likelihood transition probabilities are determined for each ethogram as described in Eq (2). Probabilities are determined for ethograms with either one, two, or three bout duration bins. The resulting matrices represent population-wide average transition probabilities. We also calculated transition matrices for individual flies, examples of which are shown in S3 Fig.

### Null model hypothesis generation

**Fig 4:**
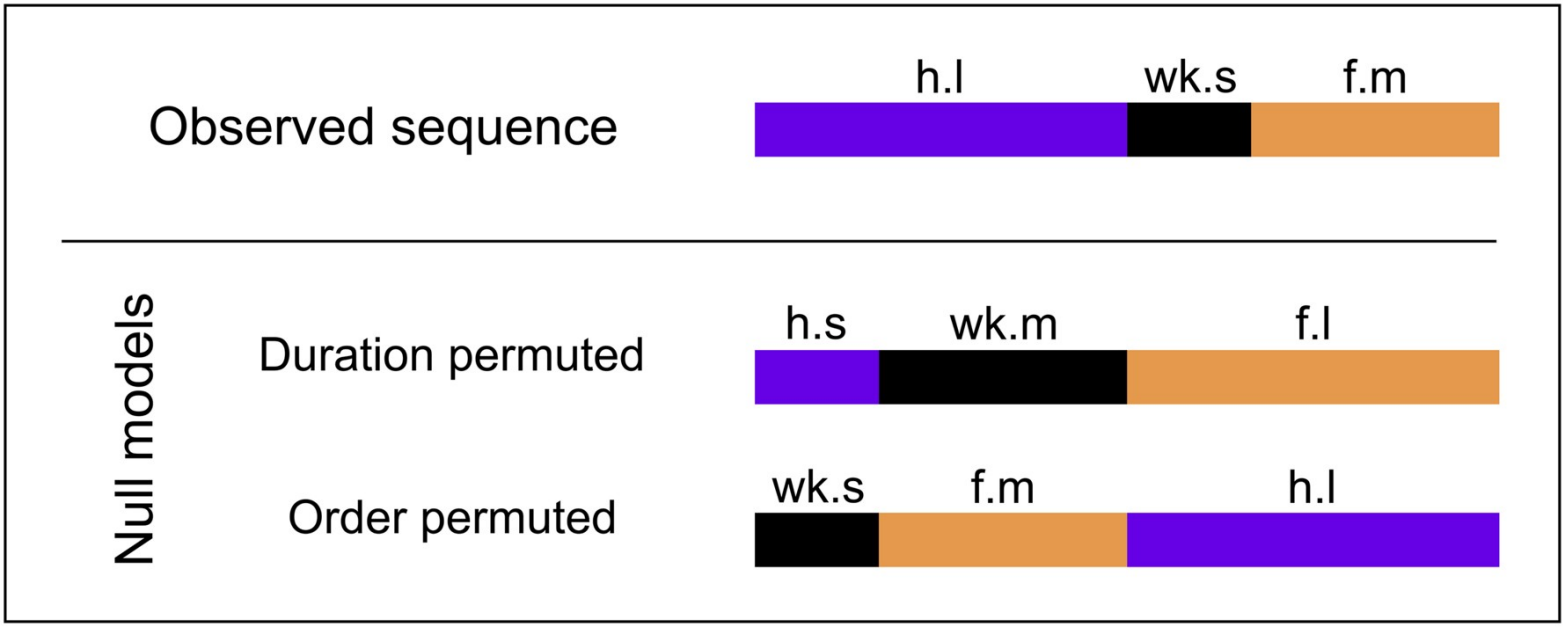
Statistical null model hypotheses (schematic). By shuffling bout duration or bout order and comparing the resultant null hypothesis transition matrices to the maximum likelihood estimate, we identify which features are statistically significant in the data and contribute to sequence structure. Shown here are schematics of the permutations we perform on the observed ethograms to generate null hypothesis transition matrices. In the duration permuted hypothesis, bout order is preserved but duration categories are randomly permuted (h = head, wk = walking, f = front leg, s = short, m = medium, l = long). In the order permuted hypothesis, bout order is permuted but the durations associated with each bout remain the same.

Statistical null models, which randomize isolated features of data while maintaining overall statistical features, provide a basis for hypothesis testing to determine the significance of results obtained from experimental data. To assess the contributions of bout identity and bout duration to sequence structure, we generate null hypotheses which independently randomize these features of the ethogram data while preserving bout duration frequency distributions within grooming action types. Null hypothesis transition probabilities are analytically tractable in the limit of infinite permutations, so we use exact formulas to calculate matrix entries (S1 Table). A representative example of each permutation on grooming bout order and duration is shown schematically in Fig 4. Our null hypotheses are as follows:

1. Duration permuted: This null hypothesis preserves action order information, but transition matrix entries are independent of duration.
2. Order permuted: This null hypothesis preserves action duration information, but transition matrix entries are independent of action order.

The resulting model fit is determined using the Bayesian Information Criterion (BIC), defined as

$$\begin{array}{c} \text{BIC}=\log\left(n\right)k-2\log\left(L\right), \end{array}$$ (3)

where *n* is the number of data points observed, *k* is the number of free model parameters, and log (*L*) is the maximized log-likelihood value. For first order Markov chains,

$$\begin{array}{c} \log\left(L\right)=\sum_{t=1}^{T-1}P\left(x_{t+1}|x_{t}\right), \end{array}$$ (4)

and

$$\begin{array}{c} P\left(x_{t+1}|x_{t}\right)={\hat{M}}_{ij}, \end{array}$$ (5)

where *i* is the row index for the behavior observed at time *t* and *j* is the column index for the behavior observed at time *t+1*.

The BIC provides a metric for evaluating model goodness-of-fit by penalizing models which use a large number of free parameters (the first term in Eq 3) and rewarding models which accurately predict unobserved data (the second term in Eq 3). Thus, models with low BIC values possess more explanatory power than models with higher BIC values but still avoid overfitting. BIC is similar to a related metric, Akaike’s Information Criterion (AIC), but penalizes models with more free parameters more severely than AIC in cases where the number of data points is much larger than the number of free parameters.

### Synthetic ethogram generation

To evaluate the ability of our models to generate sequences that are similar to our observed data, we use them to create synthetic ethograms. Synthetic sequences are generated using Monte Carlo methods within a Markov renewal process (MRP) framework. In an MRP, state transitions are governed in a manner identical to Markov chains (i.e. using a transition probability matrix). However, the amount of time spent in each state is determined in parallel by a renewal process, in which action durations are drawn from a known distribution. Here, we use the empirically observed duration distributions shown in Fig 3.

## Results

### *Drosophila melanogaster* grooming behavior progresses from anterior to posterior motifs on long time scales

**Fig 5:**
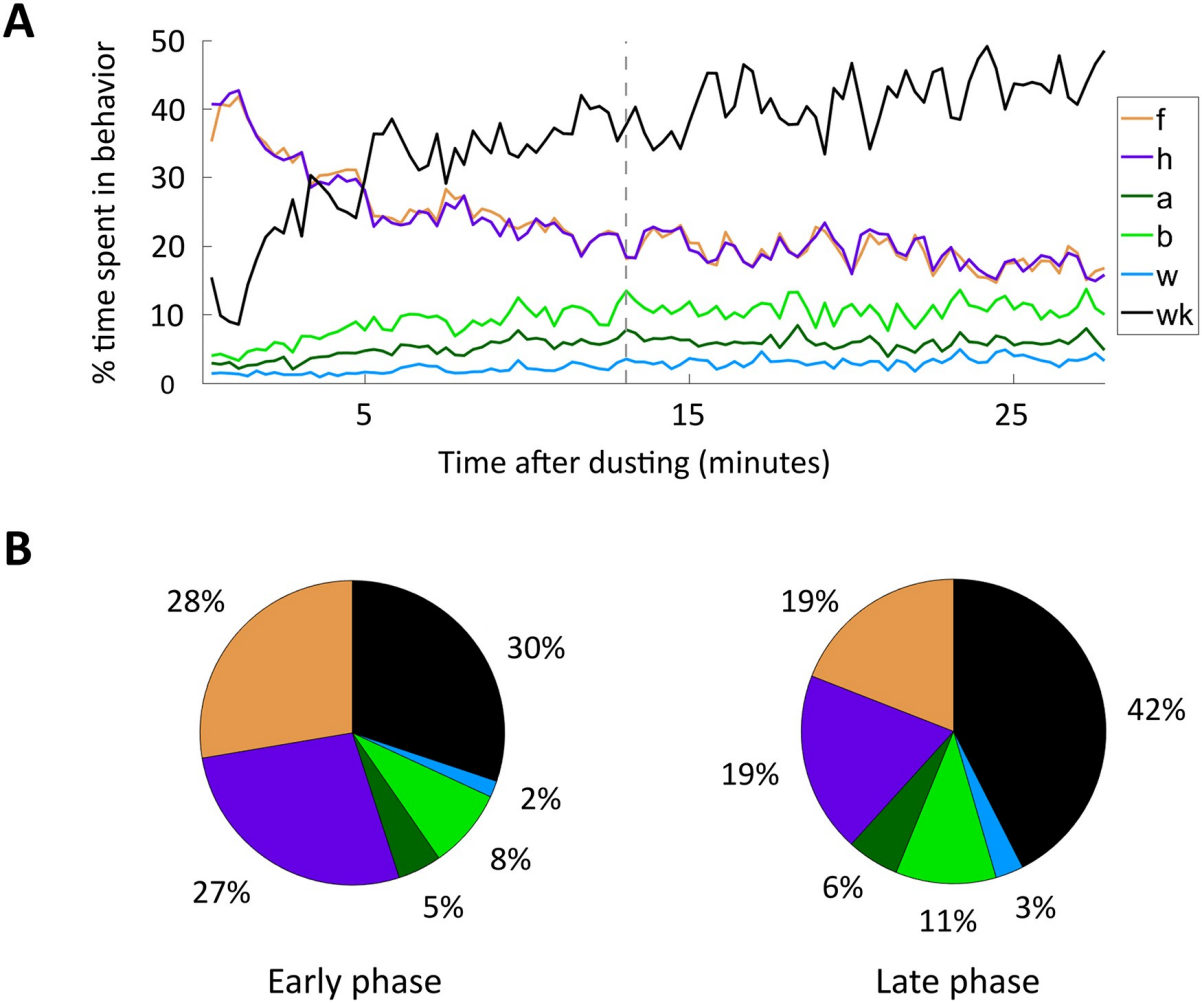
*Drosophila melanogaster* grooming progresses from anterior motif to posterior motif actions. A: Average grooming progression of *D. melanogaster* population (N = 92) after exposure to an irritant. Colored lines show the percentage of time spent in each action (f = front leg, h = head, a = abdomen, b = back leg, w = wing, wk = walking) across a 16.7 second sliding window (500 frames). Front leg rubbing and head cleaning proportions track closely with one another for the entire course of grooming. Additionally, back leg rubbing tracks closely with the sum of abdomen and wing cleaning. Dashed gray vertical line indicates the boundary between early and late grooming phases, which occurs after approximately thirteen minutes, or nearly half of the recording duration. B: Proportion of time spent in each action during the early phase (left) and late phase (right) of grooming. During the early phase, *D. melanogaster* spend the majority of time performing anterior grooming movements. In the late phase, they spend a relatively larger proportion of time engaged in posterior grooming movements and walking.

After exposure to an irritant, *D. melanogaster* grooming begins with an initial phase of mostly front leg rubbing and head cleaning (f, h). We refer to these behaviors as anterior motif grooming actions. After roughly 13 minutes, flies reach an approximate steady state consisting of heightened amounts of abdomen cleaning, back leg rubbing, and wing cleaning (a, b, w). Together, these actions constitute the posterior motif. Flies also exhibit an increased proportion of walking (wk) during the late phase of grooming. Our result, visualized in Fig 5 corroborates a similar finding reported by Seeds et al. [9] but utilizes a much larger, automatically annotated data set.

### Grooming bouts possess characteristic duration structure

We find that grooming bouts possess action-specific duration distributions. Fig 3 depicts this result. We find that anterior motif grooming actions possess remarkably similarly shaped action duration profiles, with peaks in their probability functions near 1 s. In contrast, posterior motif actions tend to be shorter. Most notably, abdomen and wing cleaning exhibit sharp distribution with peaks near 250 ms, whereas back leg rubbing exhibits a smoother distribution which more closely resembles those of anterior motif actions.

Interestingly, bouts durations are not normally distributed, suggesting a mechanism other than random generation of durations around a mean value. Instead, anterior motif duration distributions possess “shoulders” on each side of the central peak, suggesting that dividing actions into bout duration categories is not an unnatural distinction. Moreover, these “shoulders” are relatively broad due to the fact that grooming bouts consist of many quanta of individual leg sweeps (in the case of body-directed actions) or rubs (in the case of leg-directed actions), which are cyclical in nature and last approximately 150 ms per cycle. Due to video frame rate resolution limitations, we do not consider this extremely fast behavioral scale here. In this analysis, we highlight results which divide grooming actions into three duration categories in order to provide an intuitive demarcation between “short”, “medium”, and “long” actions. However, it is important to note that our conclusions are not strictly dependent on this choice of categorization scheme, as our results hold for higher numbers of duration categories as well S2 Fig.

### Intra-motif grooming actions are tightly coupled

We find that the vast majority of transitions occur within grooming motifs. Approximately 88% of transitions from anterior motif grooming bouts are to another anterior motif behavior, while about 70% of transitions from posterior motif bouts are to another posterior motif behavior. This tight intra-motif coupling is illustrated in Fig 5, as the amount of front leg rubbing tracks the amount of head cleaning extremely closely. The amount of back leg rubbing also tracks the sum of abdomen and wing cleaning very closely. This result is not affected by the choice of sliding window, as we observe a similarly strong correlation even when using a 1.67 s (50 frame) window instead of the 16.7 s (500 frame) window used to illustrate the progression shown in Fig 5.

**Fig 6:**
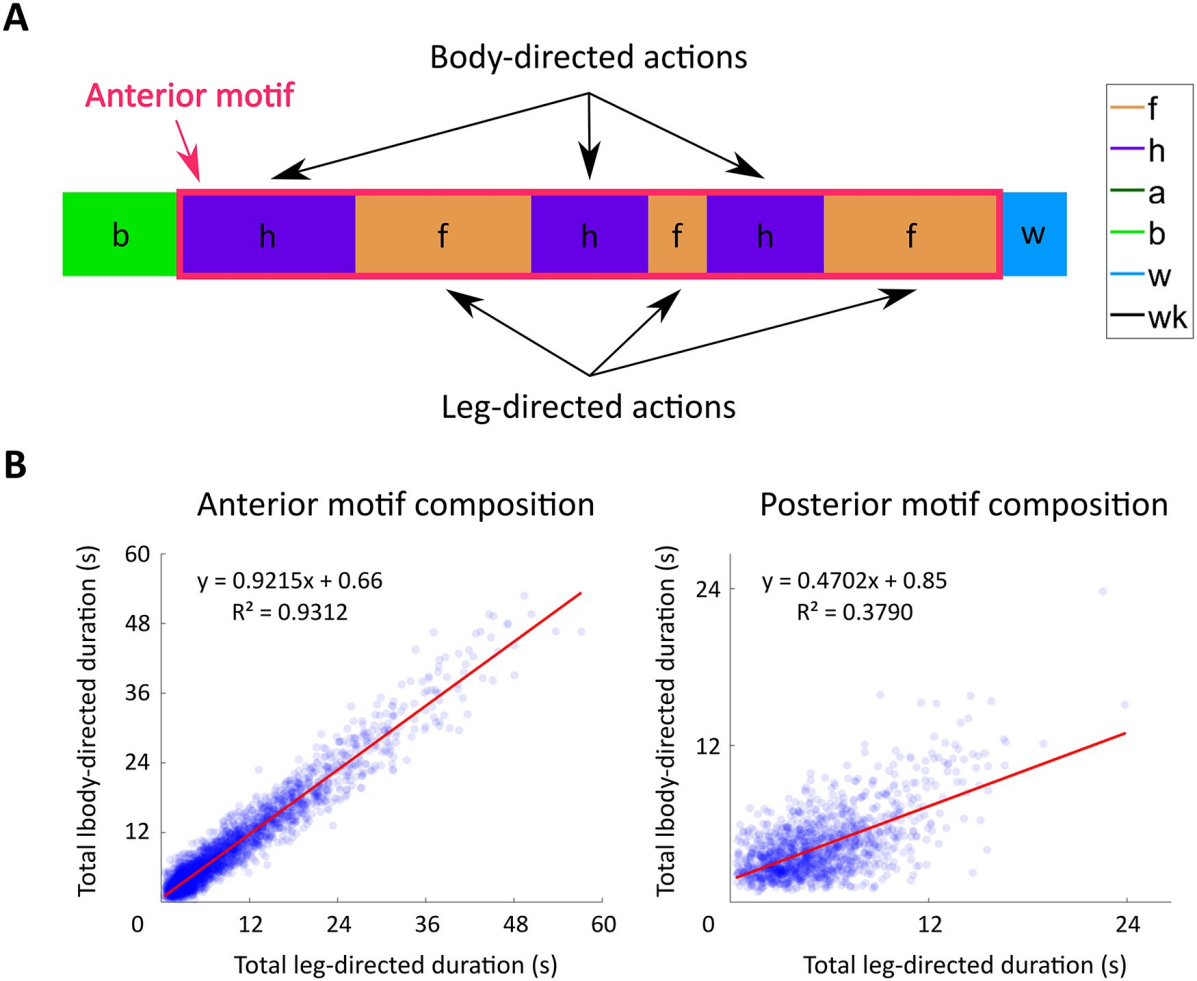
Anterior motifs are composed of highly correlated amounts of body-directed and leg-directed actions. A: Schematic of an anterior motif. Anterior motifs are defined as continuous consecutive bouts of head grooming (h) or front leg rubbing (f) flanked by non-anterior motif actions on either side. Posterior motifs are defined similarly, but consist of abdomen grooming, back leg rubbing, and wing grooming (a, b, and w). Both motifs contain body-directed actions (h, a, and w), which clear irritant from the body. Leg-directed actions (f and b) clear irritant from the legs, which collect irritant during body-directed grooming actions. In this example, the anterior motif consists of six grooming bouts of varying duration. B: Anterior motifs exhibit a strong linear relationship between the total amount of time spent performing body and leg-directed actions (left). Each point corresponds to an observed motif consisting of four or more actions. Posterior motifs (right) display a weaker linear trend with greater amounts of leg-directed grooming.

We find that this strong coupling emerges from the structure of individual grooming motifs. Fig 6 provides an illustrated example of the structure of an anterior motif. We find that, across flies and independent of the time after grooming, anterior motifs contain nearly perfectly correlated amounts of body and leg-directed grooming. This strong correlation is somewhat surprising given that consecutive anterior grooming bouts exhibit a non-linear relationship (S4 Fig).

**Fig 7:**
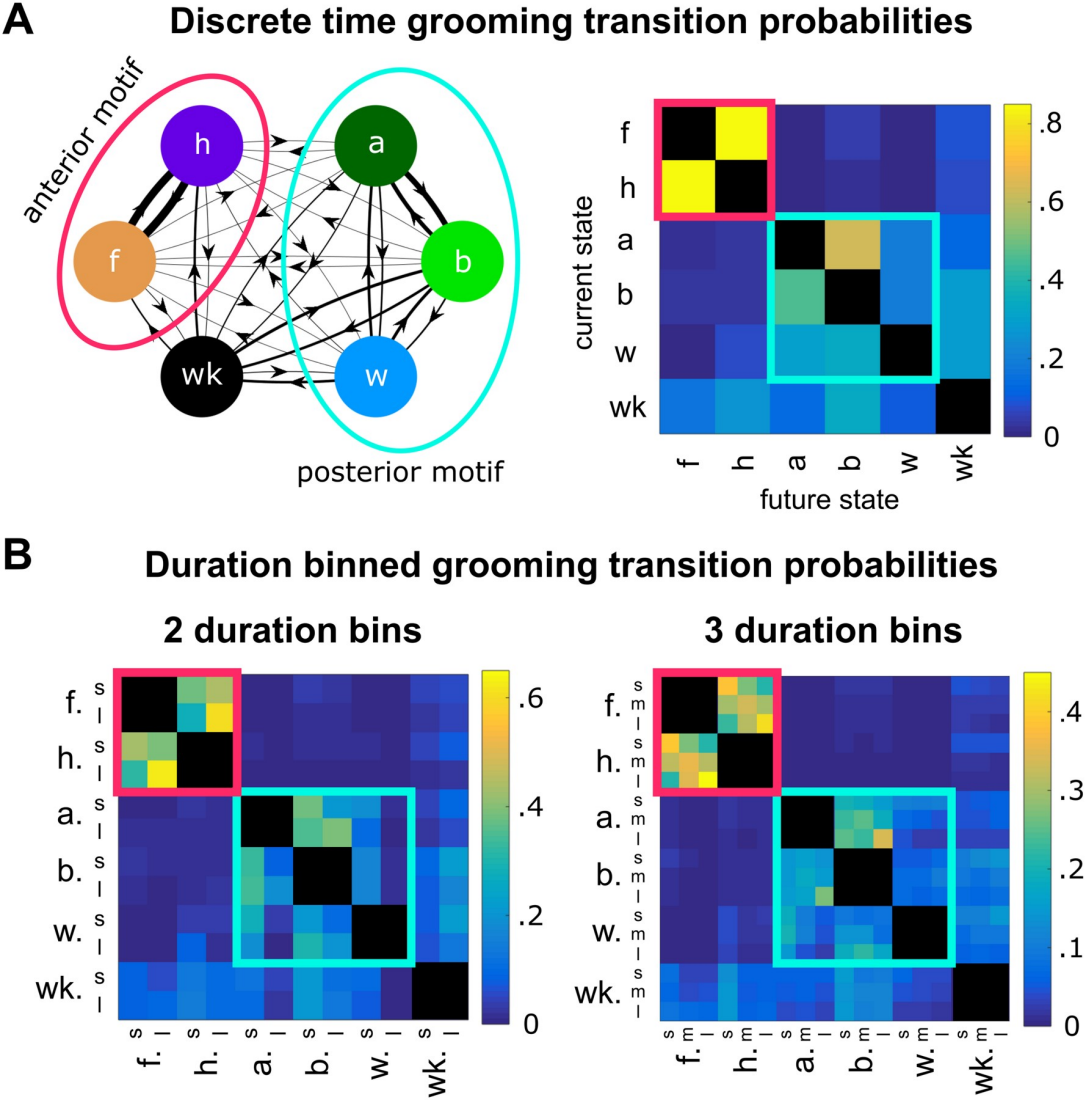
Within-motif transitions dominate grooming syntax. A: The maximum likelihood transition matrix, $\hat{M}$ (Eq 2), provides the best estimate of the Markov chain dynamics observed in grooming behavior (Eq 1). The network representation of $\hat{M}$ (left) illustrates transition probabilities using edge thickness (thicker edges indicate higher probabilities). Shown on the right is the matrix representation of discrete time transition probabilities (fit to the discrete time ethogram from Fig 1 with no duration information), with probability magnitude indicated by color. Here, the anterior motif, consisting of front leg rubbing and head cleaning, is indicated by the red circle (left) and red square (right). The posterior motif, consisting of abdominal grooming, back leg rubbing, and wing cleaning, is delineated by the blue circle (left) and blue square (right). B: Maximum likelihood transition probabilities for ethograms binned according to the schema shown in Fig 3. Matrices fit to data with two (left) and three (right) duration categories show similar within-motif structure with increased resolution (s = short, m = medium, l = long). For example, both matrices illustrate that anterior motif transitions between long bouts dominate syntax.

This tight intra-motif coupling is also reflected in the structure of Markov transition matrices fit to our behavioral data (ethograms). We fit a first order discrete time Markov chain to our data and the resulting matrix, denoted by $\hat{M}$ (Eq 2), contains the maximum likelihood estimates of the probabilities of transitioning from one grooming action to another. Fig 7 depicts the structure of these transitions in both network and matrix form, illustrating the transition probabilities used to define the Markov chain in Eq (1). In Fig 7, anterior and posterior motif grooming actions are indicated by light red and blue outlines, respectively. Note that self-transitions are explicitly prohibited as a result of the ethogram discretization method. Consequently, the network diagram lacks self-loops and all diagonal elements in transition matrices are blacked out, signifying zero value entries.

### Grooming bout duration contributes to sequence structure at short time scales

**Fig 8:**
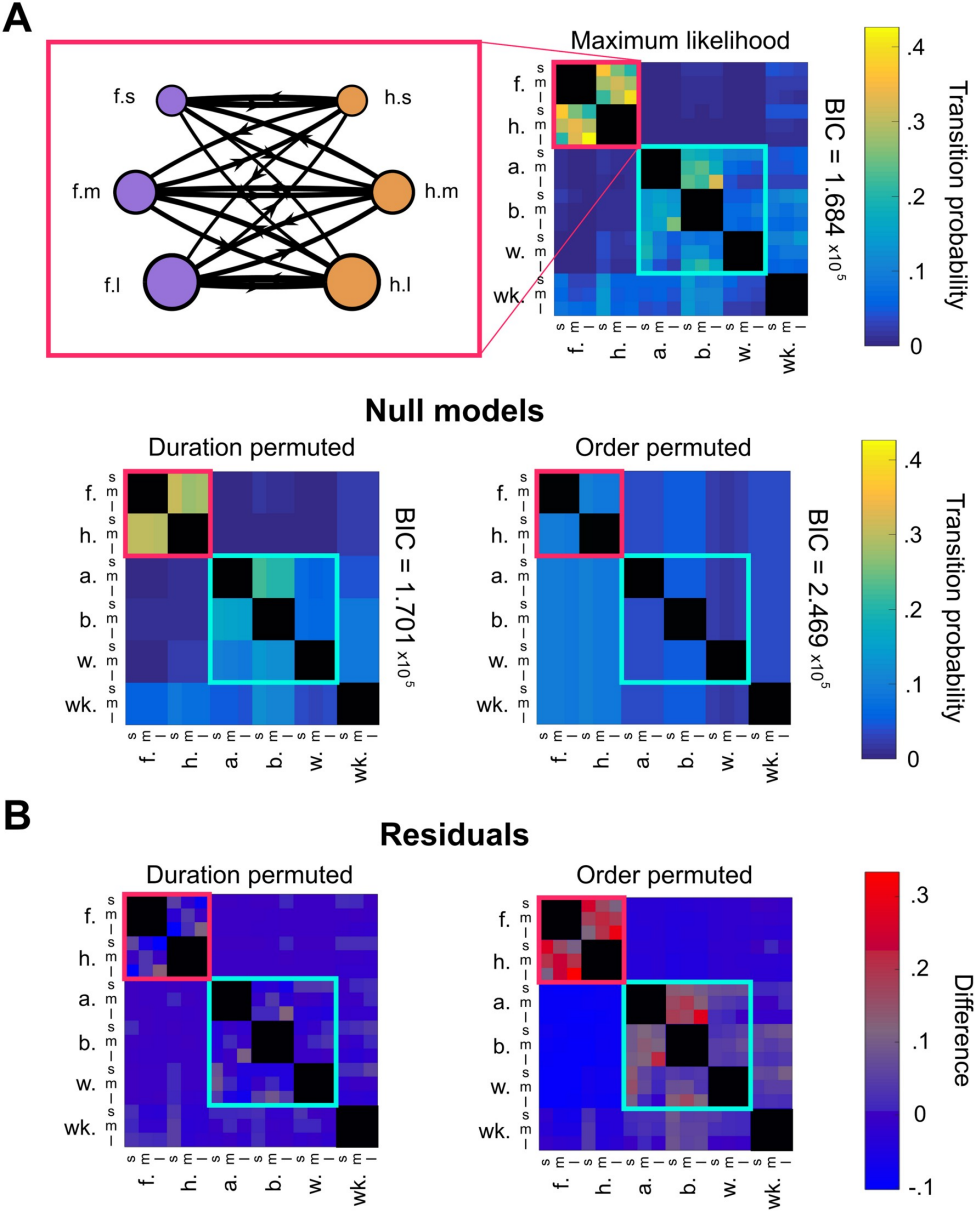
Grooming bout duration contributes to syntax at the scale of individual transitions. A: Probabilities from the maximum likelihood transition matrix (top) differ significantly from null hypothesis transition matrices (bottom) to varying degrees, indicating that both grooming action order and bout duration contribute to grooming syntax. Shown on the top left is the network visualization of anterior motif transitions, with edge withs proportional to transition probabilities. Notice that transitions between actions belonging to the same duration category possess relatively high probabilities and appear nearly symmetric, suggesting a coupling mechanism between anterior motif actions. BIC values (right of matrices) provide validation that the maximum likelihood model captures statistically significant features of the data. The maximum likelihood transition matrix has a lower BIC value than the null hypotheses used for comparison, indicating that both bout order and duration contribute to sequence syntax. B: The residual values, or the difference between the maximum likelihood matrix and the null model matrices, illustrate that specific transitions differ from what the null hypotheses would predict. The duration permuted null model matrix exhibits block structure but fails to capture the temporal relationship between bouts, as illustrated by the red values in several “long-to-long” transitions, for example. The order permuted null model differs even more severely, indicating that, while duration dependence plays a role in sequence structure, action order is still the primary determinant.

In Fig 7, we illustrate the fine-grain temporal structure present within the identified motifs. Transition matrices with either two (left) or three (right) duration bins reveal the presence of certain high-probability transitions. Most notably, we observe that transitions between long anterior grooming actions dominate to an extent not found in the null hypotheses we use for comparison. For example, when using three duration categories, the probability of transitioning from a long front leg rub to a long head cleaning bout is ∼39%. This is significantly higher than the corresponding transition probabilities in the duration permuted and order permuted null hypotheses, which are ∼28% and ∼13% respectively. These differences are depicted in greater detail in Fig 8.

Additionally, we observe that, regardless of the number of duration bins, the anterior motif transition probability structure is nearly mirror symmetrical along the diagonal (i.e. transitions between front leg rubbing and head cleaning bouts have the same probabilities). In contrast, posterior motif transitions are less strongly symmetrical, with transitions between abdomen cleaning and back leg rubbing exhibiting the highest probabilities (Fig 7).

We find that the probabilistic rules describing transitions between consecutive bouts depend on two features of immediate behavioral history: previous bout identity and previous bout duration. To validate this observation, we evaluate the quality of the maximum likelihood model using the Bayesian Information Criterion (BIC). This metric, which is described mathematically by Eq 3, rewards models that produce accurate predictions and penalizes those that contain more parameters. The model with the lowest BIC score is considered the best fit to the observed data. Using BIC, we show that, compared to statistical null model hypotheses, the maximum likelihood transition probability best describes the data without overfitting. BIC values and null hypothesis matrices used for comparison are shown in Fig 8. Since each null hypothesis matrix contains fewer free parameters than the maximum likelihood model, the advantage of the maximum likelihood matrix in BIC is due entirely to its superior explanatory power.

Inspection of BIC values indicates that bout order is the strongest individual determinant of grooming syntax and that bout duration provides an additional contribution. Fig 8 shows the BIC values for each matrix as determined using data binned into three duration categories. The order permuted null hypothesis possesses a significantly larger BIC value than the duration permuted null hypothesis, indicating that disrupting bout order degrades predictive power most significantly.

By construction, each null hypothesis transition matrix contains identical row structure within sets of rows corresponding to the same grooming action. However, only the duration permuted transition matrix possesses block structure similar to that found in the maximum likelihood matrix, as seen in Fig 8. This feature also reinforces the claim that action order provides a relatively larger contribution to syntax, since preserving action order results in the preservation of transition matrix block structure.

### Bout-to-bout transitions are largely stationary despite changing sensory conditions

**Fig 9:**
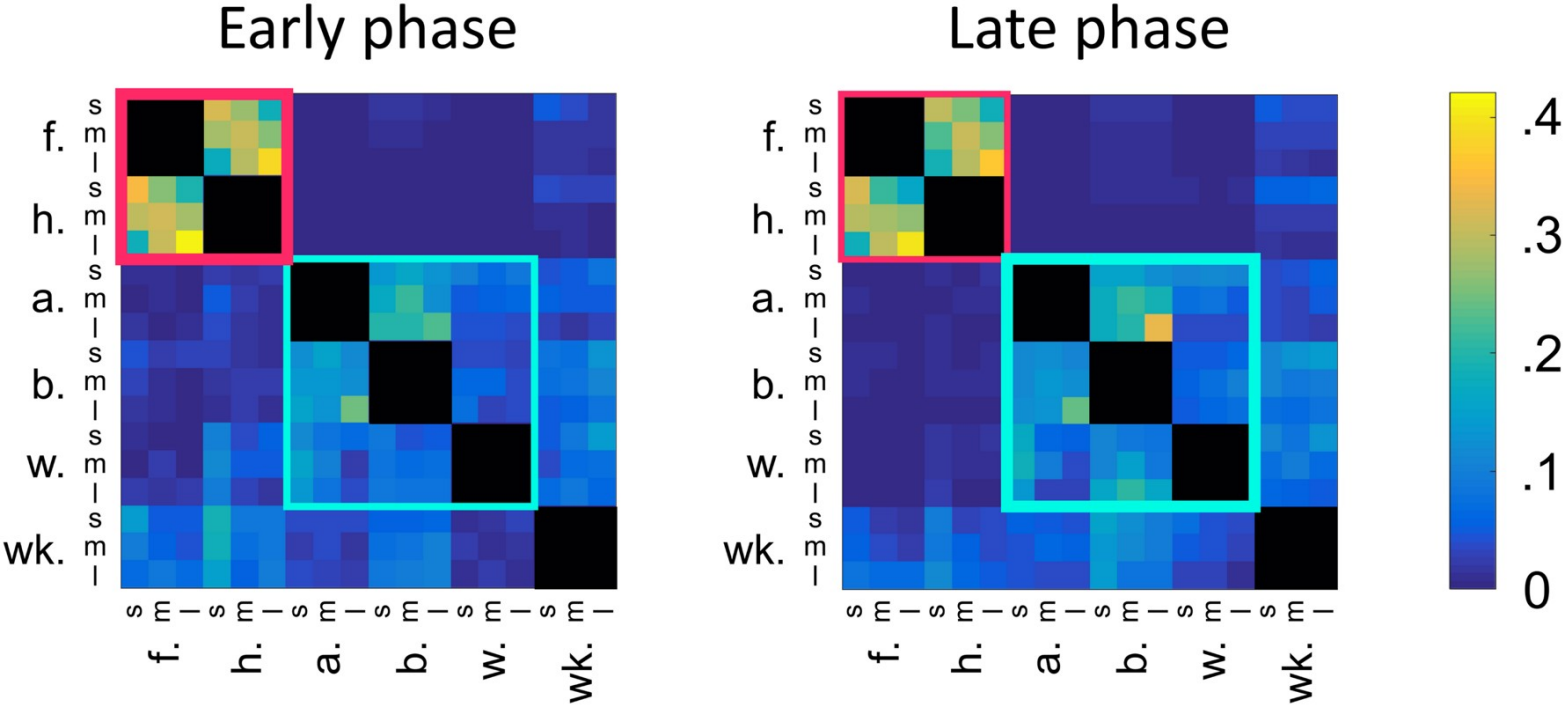
Transition probabilities are largely stationary across the entire grooming progression despite changing sensory conditions. Shown here are the population average transition probabilities for the early phase (left) and late phase (right) of grooming. The border between these phases is indicated by the dashed gray line in Fig 5. In the early phase, flies prioritize anterior grooming motifs, indicated by the thick red outline. In the late phase, flies perform more posterior grooming, indicated by the thick blue outline. We observe that transitions between front leg rubbing and head cleaning bouts exhibit consistent duration dependence regardless of when they occur in the sequence. Posterior motif transitions display similarities as well, but transitions between long abdomen grooming bouts and long back leg rubbing bouts are significantly more likely late in grooming. Overall, the relative stationarity of these transition probabilities despite changing sensory conditions suggests the existence of an internal mechanism that dictates bout durations. However, sensory stimuli also appear to play a role in modulating grooming transitions on long time scales, as transitions from posterior motif actions to anterior motif actions become less likely in the late phase.

Fig 9 shows the average transition probability matrices with three duration categories for the early and late phases of grooming, as separated by the dashed line in Fig 5. Data were separated into early and late phase data for each fly and maximum likelihood transition probabilities were determined as described in Methods and Models. Here, we determine the boundary between phases to be the average time elapsed after flies have performed half of their discrete grooming actions. We also conducted a similar analysis in which we fit transition matrices only to the first and last third of data so as to avoid using the middle portion of the progression. This did not alter our results (S5 Fig).

Qualitatively, these matrices exhibit strong similarities, despite the differences in grooming proportions, as shown in Fig 5. In particular, transitions between anterior motif grooming movements are similar across phases of the recording. Additionally, we find that 253 of the 266 (95.1%) non-zero entries change by less than 5% and 168 (63.2%) entries change by less than 1% (S6 Fig). This suggests that the rules for sequence generation are nearly fixed, in spite of changing sensory input. Most notably, transitions between long abdomen grooming bouts and long back leg rubbing bouts are enriched in the late phase of grooming, illustrating that grooming rules, while similar, are not completely stationary over time.

### A slowly varying model captures grooming syntax at long, intermediate, and short time scales

Markov models are steady state models and, as such, only exhibit equilibrium dynamics when used in a generative manner to create synthetic data. The first order maximum likelihood Markov model presented above is best suited for describing the late stages of grooming, which resembles steady state behavior. This is an obvious shortcoming of using a Markovian framework for analyzing the ethograms presented here, as flies display a dynamic progression (Fig 5), likely due to changing amounts of irritant over the course of grooming.

In order to more explicitly model these changing sensory dynamics, we utilize a nonstationary, or time-varying, Markov renewal process (MRP). Here, we introduce a nonstationarity in the form of a time-varying transition probability matrix. In order to maintain as parsimonious a model as possible, we define the transition probability matrix as a time-dependent convex combination of an early phase matrix and a late phase matrix (Eq 6). The early phase matrix is determined using maximum likelihood fitting on only the first 200 actions of each fly. Likewise, the late matrix uses each fly’s final 200 actions. This ensures that early and late phases are well-separated, as each ethogram averages approximately 1000 bouts.

The time-varying transition probability matrix **M**(*t*) used in the MRP is defined as

$$\begin{array}{cc} M(t) & =\{\begin{array}{cc} \frac{13-t}{13}M_{early}+\frac{t}{13}M_{late}, & t<=13,\\ M_{late}, & t>13 \end{array} \end{array}$$ (6)

where *t* is in minutes. After 13 minutes, the late phase transition matrix is used due to the observation that flies exhibit steady state behavior after approximately that amount of time. Bout duration distributions used in the MRP do not change over time, as we did not find any significant difference between early and late bout durations (S7 Fig).

**Fig 10:**
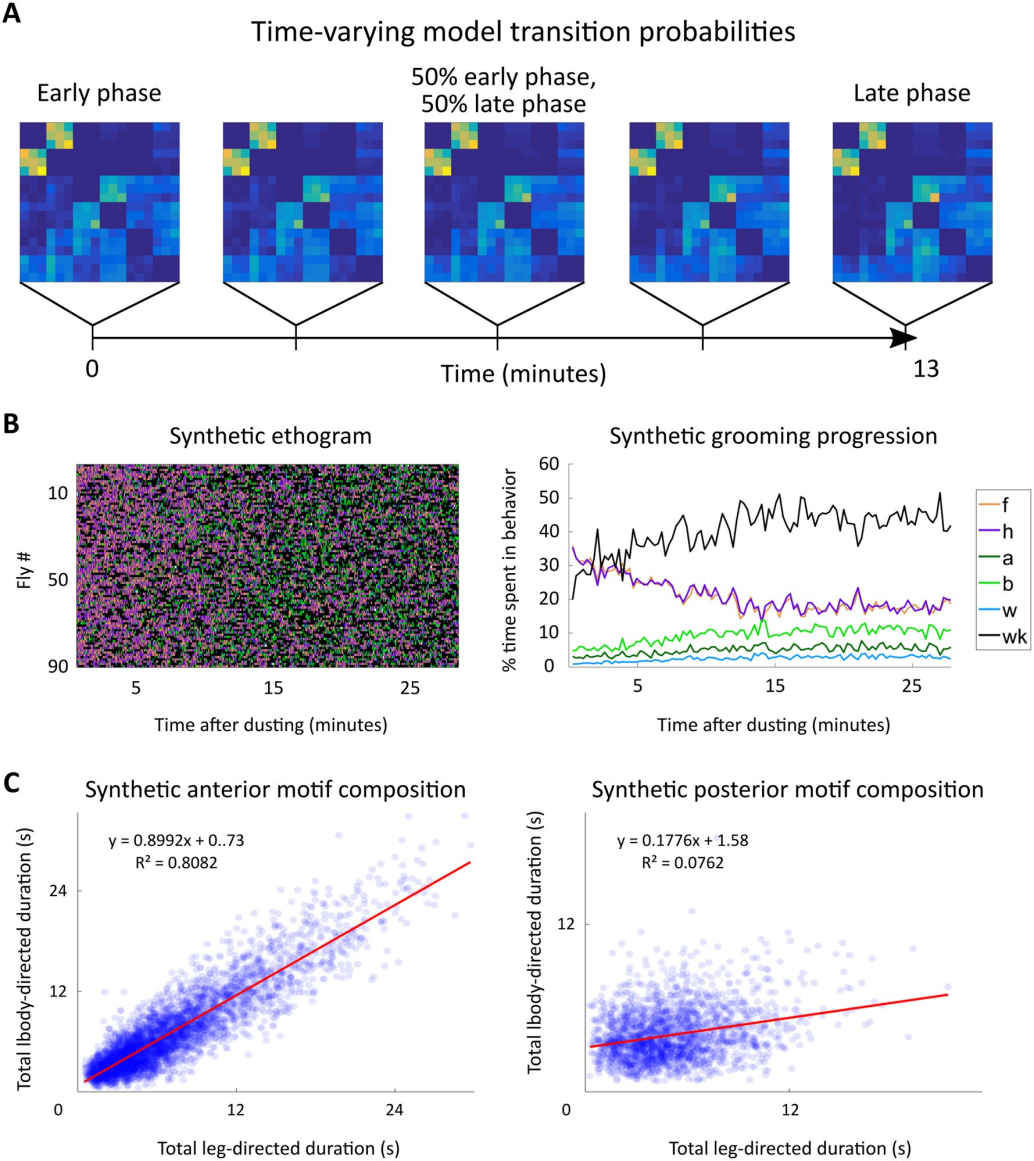
A nonstationary Markov renewal process recapitulates grooming progression and bout structure. A: Illustration of the time-varying transition matrix, **M**(*t*), used for generating synthetic ethograms. To approximate changing sensory conditions in the simplest possible manner, two transition matrices, **M**_*early*_ and **M**_*late*_, are fit to the first and last 200 actions in the discrete time ethogram with 3 duration categories, respectively. As time evolves in the synthetic ethogram simulation, **M**(*t*) changes as described in Eq 6. At the beginning of the simulation, **M**(*t*) is identical to **M**_*early*_ and after 13 minutes, it is identical to **M**_*late*_. Between those times, it is a linear combination of the two matrices. B: Synthetic ethograms display the characteristic progression from anterior to posterior grooming, as seen from comparison with Fig 5. C: Synthetic ethograms reproduce observed anterior motif composition. Synthetic anterior motifs (left) exhibit a similar, though slightly weaker, trend as observed in our data (Fig 6). Posterior motifs are less similar, indicating that other factors may be necessary to explain posterior motif structure.

Surprisingly, using this simple linear interpolation as a proxy for changing sensory conditions yields a model which produces ethograms that closely resemble our observed data (Fig 10). This interpolation serves as a first-order approximation to changing sensory drive, as flies constantly remove dust at an unknown rate over the course of grooming. Synthetic flies prioritize anterior grooming early on and gradually transition into a late phase which prioritizes posterior grooming and walking. This synthetic progression closely matches the actual progression shown in Fig 5. The synthetic ethograms also exhibit a tight coupling between the amount of anterior grooming actions over time. Moreover, synthetic motifs possess similar structure to observed motifs, as shown in panel C of Fig 10. Finally, the use of empirical transition matrices and bout duration distributions in our MRP guarantees that synthetic ethograms possess the same duration dependence at short time scales as actual data.

## Discussion

### *Drosophila melanogaster* respond to irritant by executing structured, modular sequences of cleaning movements

After exposure to an irritant, *D. melanogaster* engage in a series of grooming actions in order to clear the irritant from their body. These movements are highly non-random, as indicated by their structure at three separate temporal scales. On the longest time scale, grooming progresses over several minutes from an early anterior-heavy phase to a later phase which contains elevated levels of posterior-directed actions and walking as illustrated in Fig 5. At an intermediate time scale, anterior and posterior grooming motifs exhibit strong correlations between the amount of body and leg-directed movements present in each motif, as shown in Fig 6. Finally, grooming syntax exhibits non-random structure at the short time scale of individual grooming bout decisions, as illustrated by the non-random structure of transition matrices shown in Fig 7. Together, these findings suggest that there are distinct syntactic rules that govern grooming sequence structure at different temporal scales.

Here, we isolate short time scale grooming syntax by using a binning scheme to represent grooming bouts as semi-discrete actions (e.g. short, medium, or long bouts). Across different binning schemes, within-motif transitions (i.e. anterior-to-anterior or posterior-to-posterior) dominate syntax at the scale of consecutive bouts, as indicated by the block-like structure of transition matrices in Fig 7. The likely functional explanation for this tight correlation is that *D. melanogaster* engage in leg-centric grooming as a way to clear irritant that has collected on their limbs after body-centric grooming. Past work on blowflies also suggests that this coupling may be due to postural considerations, since fewer motor actions are required to transition between movements which use the same set of legs [6].

Moreover, the amount of front leg rubbing over the course of the grooming progression corresponds nearly exactly to the amount of head cleaning performed by the flies. The amount of back leg rubbing is also directly proportional to the amount of abdomen and wing cleaning (Fig 5). Though the total amount of time spent performing actions within motifs is tightly coupled, consecutive bout durations do not exhibit a simple linear relationship (S4 Fig). Instead, transitions between body-centric and leg-centric actions of certain durations dominate. In particular, anterior motif movements exhibit nearly symmetric dynamics, with a preference for long-to-long transitions. In contrast, posterior motif transitions are asymmetric—this can be seen in Fig 7, as transitions from body-directed actions to leg-directed actions have different values than transitions in the opposite direction. This suggests a mechanism whereby internally generated dynamics that are specific to each motif guide intra-motif transitions.

### Symmetric anterior motif transition rules suggest higher-level transition frequency control

Anterior motif transitions display symmetric transition rules, as shown in Fig 7. The transition from head cleaning to leg rubbing may exhibit a strong correlation in duration for the following reason—the longer the head cleaning bout, the more particulate the legs accumulate, which subsequently requires more time to clear. However, it is more surprising that after performing a bout of leg rubbing the animal should display a similar duration preference upon transitioning back to head cleaning; once the front legs are sufficiently clean, we would not expect the animal to exhibit any duration preference on the subsequent bout. Additionally, the bout duration distributions for grooming actions remain strikingly similar across the entire recording, suggesting that sensory considerations do not account for the duration dependence we observe (S7 Fig).

These observations support the hypothesis that alternations between anterior grooming bouts are regulated by a common source or common dynamics that dictate intra-motif transition frequency. If this is the case, future investigation of neural circuitry could focus on identifying neural activity that oscillates in phase with grooming bout alternations. Under this model, flies would then need to combine discrete decisions about which motif to perform with continuous decisions about how long to maintain a motif. This provides other targets for future experiments, as we could look for neural activity corresponding to transitions between motifs, explore manipulations that induce transitions between motifs, and search for factors that affect the duration of motifs.

### Modeling suggests that sensory input modifies internal grooming programs

Although sensory input is sufficient to initiate individual grooming bouts, it has been difficult to assess the role of sensory input in grooming on longer time scales, since direct quantification of irritant relies on invasive protocols [25]. To date, the contribution of internal dynamics to grooming sequence generation also remains unknown. The discovery of duration dependence in grooming suggests that flies do not rely exclusively on sensory information to make grooming decisions, leaving open the possibility that sensory input modifies pre-existing autonomous grooming programs.

Here, we abstract the contribution of sensory input, incorporating it as a parameter in a time-varying Markov renewal process. Specifically, we approximate changing sensory conditions as a linear phenomenon which dictates the degree to which flies favor early versus late grooming rules (Fig 10). This choice of nonstationarity preserves the parsimony of our MRP, as it precludes the necessity of re-calculating transition probabilities over time. Additionally, the similarity between early and late intra-motif transition probabilities (Fig 9) provides some leeway for the choice of nonstationarity, bolstering our confidence that a simple linear rule is sufficient to approximate true sensory conditions.

Since our MRP reliably reproduces true ethogram statistics without explicitly modeling sensory input, we propose that, rather than acting in a purely reflexive manner, *D. melanogaster* instead use sensory information to modulate internal grooming programs. This type of slowly varying process has been observed in other organisms, suggesting that it is a useful framework for describing sequential behaviors [26]. Additionally, this hypothesis can be tested in future experiments which manipulate sensory conditions using irritants with different properties or optogenetic stimulation of sensory receptors.

### A role for sensory information-independent transition rules in grooming

From a computational perspective, sensing is costly when it requires high-frequency updates and can provide readouts at a high resolution, as specialized neural circuits must be formed, maintained, and integrated in order to provide accurate, real-time readouts. This can lead to increased metabolic costs on several fronts, as increased neural activity and behaviors intended to protect and maintain sensory machinery can also require energetic resources [27]. In contrast, autonomous internal dynamics require only sparse sensory updates and may not require the same level of neural activity as is needed for sensory processing. If sensory drive alone, triggered by the presence of an irritant, dictates sequence generation in a purely reflexive manner, competing priorities may lead to rapid alternations between actions without any distinctive duration structure, consistent with neural circuity implementing competitive inhibition with a transition cost [9].

During the execution of a grooming motif, frequent, high-resolution sensory updates may not be necessary. Instead, the moment-to-moment decisions about execution of grooming bouts can be automated by utilizing patterned internal dynamics. Several models of behavioral generation in which internal triggers drive sequence generation have been proposed [28, 29]. Since we show that intra-motif transitions exhibit bout duration-dependent structure, we suspect that internal triggers may provide a basis for transitions on short time scales after a discrete grooming motif decision is made. We consider this a strong possibility due to the fact that anterior motif transition probabilities are nearly stationary across grooming even though the dust distribution on the body is constantly changing. The near stationarity of these rules in spite of a changing stimulus suggests to us that behavior possesses an internally generated, fixed component. The discovery that grooming progresses gradually from anterior to posterior movements suggests that, on long time scales, *D. melanogaster* utilize sensory information to dictate decisions about which grooming motif to perform, since the dust distribution changes over the period of cleaning.

In discussing temporal correlations between neural activity across spatial scales, Berman et al. [10] note that “(a)lthough no such correlation has been specifically found in *Drosophila*, our results suggest that such neuronal patterns may exist: perhaps by combining descending commands from the brain with local circuitry within and emerging from the ventral nerve cord.” This observation is consistent with our interpretation of our results, as local circuitry in the ventral nerve cord could plausibly generate internal dynamics which introduce duration dependence to action selection.

By combining sensory input and internal dynamics, *Drosophila melanogaster* nervous systems may utilize multi-level control algorithms which make discrete, “ballistic” decisions about the onset and type of continuous behaviors, allowing them to update infrequently at low sensory resolution. Once the decision is made, the execution of the behavior can be guided primarily by autonomous internal dynamics. In flies, these internal dynamics may not be unique to grooming, as freely-behaving *D. melanogaster* exhibit hierarchical Markov-like behavior in the absence of external sensory stimuli [10, 18]. Additionally, the identification of stereotyped subroutines in *D. melanogaster* locomotion further supports the idea that specialized neural circuits can automate and reproduce portions of behavior, reducing the need for constant calibration via sensory feedback [19]. We can further explore this idea by studying whether spontaneously grooming flies or those stimulated using optogenetic methods exhibit similar syntactic rules.

In larval and mature *D. melanogaster*, specialized, isolated neural circuits known as central pattern generators (CPGs), use rhythmic activity to guide execution of locomotion and courtship song sequences [30, 31]. In humans, CPGs are believed to facilitate chewing and breathing subroutines, such as individual jaw or pharyngeal movements [32, 33], indicating that such neural circuit elements are common and flexible enough to carry out a wide variety of functions.

Since CPGs can modulate motor programs independently of sensory input, they are a natural candidate to execute grooming behavior on the scale of individual leg sweeps and rubs within a grooming bout. We do not explicitly model behavior at this temporal scale, but the ABRS classifier applied to video at the frame rate used here identifies actions nearly at the temporal resolution of individual leg movements. We plan to extend analysis to this time scale in future work.

Additionally, robotic systems that utilize multi-layer CPG architectures can carry out locomotor tasks, indicating that hierarchical CPG structures can generate sequential behaviors from smaller elements [34]. Recent work indicates that circadian rhythms and genetic factors contribute to *D. melanogaster* grooming behavior as well, leaving open a role for internal regulation of sequence generation at longer time scales [35].

### Conclusion

When describing complex behavioral phenomena, formal mathematical models can bridge the gap between phenomenology and biological mechanisms by providing parameters that may correspond to underlying neuronal activity. In many cases, statistical model parameters do not directly describe to underlying biological structures, but data analysis can suggest hypotheses for future exploration.

Here, we analyze large-scale automatically annotated data sets to reveal the syntax of *D. melanogaster* grooming. Using Markov models to detect features of grooming sequences, we find that grooming actions exhibit duration dependence at the scale of individual bouts and grooming motifs. Then, we produce realistic synthetic ethograms by introducing a slow modulation of grooming rules meant to abstract the contribution of sensory input. Together, these findings suggest that internal programs dictate grooming decision on fast time scales but are modulated by sensory input over longer time scales.

As the tools for computational analysis of behavioral data continue to develop, interdisciplinary approaches that use mathematical tools to illuminate biological mechanisms will yield further insight into the generation of sequential behavior. Future experiments involving the manipulation of sensory experiences using optogenetic stimulation or other methods will also allow researchers to test hypotheses with unprecedented precision and scope. Together, these advances promise to improve our understanding of sequence generation by connecting mathematical descriptions of behavior with the underlying neural circuitry, as we are now motivated to search for circuits controlling long, intermediate, and short time scale grooming rules and determine how they interact with sensory circuits. Finally, our work suggests that temporal dynamics can and should be included when using statistical models to assess complex behaviors.

## Supporting information

S1 Fig Machine classifier performance is similar to human classification accuracy. A: Here, grooming behaviors are visualized in low-dimensional space using t-SNE, which preserves local distances between nearby points. Color indicates behavior type. Behaviors are well-separable by type. B: Example classifications of ethograms of grooming behaviors are shown. Human-algorithm agreement is about 75% (in terms of number of matching frames).
(TIF)

S2 Fig Transition probability matrices exhibit duration-dependent structure across many choices of binning schemes. When transition probabilities are calculated from ethograms with 4 (left) or 5 (right) duration categories, fine-grain temporal structure is still present (compare the matrices shown here to those shown in Fig 7). The existence of this trend across binning schemes ensures that duration dependence is a phenomenon present in the data and not an artifact of data processing.
(TIF)

S3 Fig Individual flies possess stable syntax that resembles the group average. Two examples of individual fly transition probabilities illustrate that, although syntax varies between individuals and over time, individuals are relatively stable and resemble the group average (compare to Fig 9). Though we did not fully characterize individual differences here, future studies will aim to examine individuals more closely.
(TIF)

S4 Fig Consecutive anterior bout durations do not exhibit a linear relationship. Plotted here is the relationship between consecutive anterior grooming action durations. Linear regression produces a poor fit, indicating that duration dependence is not linear.
(TIF)

S5 Fig Sampling early and late phases differently does not alter the observed syntax. To ensure that our choice of boundary between early and late phases does not alter our results, we partition our data in a stricter manner so that the transition period which occurs during the middle of the grooming progression is excluded. Partitioning the data into thirds produces transition matrices with structure and stationarity similar to those shown in Fig 8.
(TIF)

S6 Fig Small residuals between early and late transition probabilities indicate that most grooming rules are stationary over time. We find that 253 of the 266 (95.1%) non-zero entries change by less than 5% and 168 (63.2%) entries change by less than 1%. This was a very surprising finding and suggests that, although some non-stationarity does exist, it is much smaller than we had expected given that the average time spent performing different behaviors varies between the early and late phases.
(TIF)

S7 Fig Bout durations do not change between early and late phases. Probability density plots of bout duration distributions illustrate that bout durations remain consistent between early and late phases of grooming. Early phase data is plotted with a thick line. Late phase data is plotted with a thin line.
(TIF)

S1 Table Analytic formulas for null model transition matrices. Given in S1 Table are the analytic formulas for transition matrix entries, **M**_*ij*_, for each null hypothesis transition matrix. Here, *i* denotes the row and *j* denotes the column of **M**. Note that, in the three duration category case, each state has both an action and duration identifier (e.g. row h.s. corresponds to a head cleaning action in the short duration category). *P*(*x*_*action*,*j*_|*x*_*action*,*i*_) is the absolute probability of transitioning from the grooming action represented by row *i* to the action represented by row *j*, regardless of duration (this is given by the discrete time transition probability matrix in Fig 7A). *P*(*x*_*duration*,*j*_) is the absolute probability of the grooming action represented by row *j* belonging to the duration category represented by row *j*. *P*(*x*_*j*_) is the absolute probability of state *j*.
(TIF)
