# Supplementary figures and images for: Drosophila melanogaster grooming possesses syntax with distinct rules at different temporal scales
Source: PLoS Comput Biol. 2019 Jun 26;15(6):e1007105. doi: 10.1371/journal.pcbi.1007105 (PMC6594582; doi:10.1371/journal.pcbi.1007105)

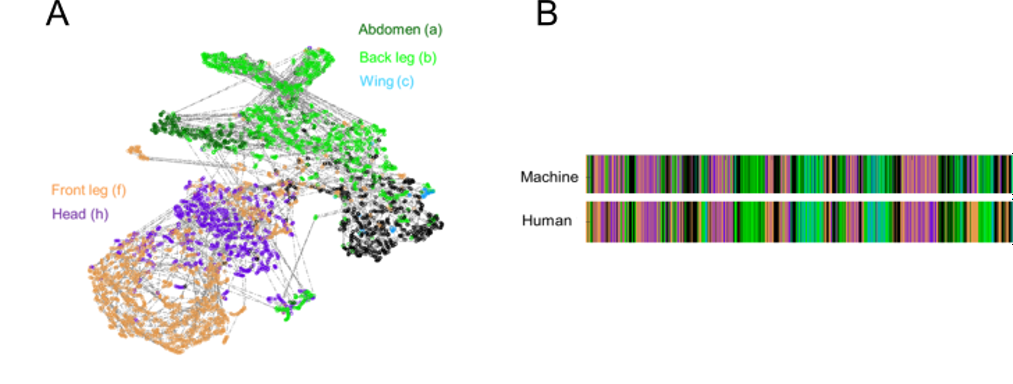

Supplement: S1 Fig — A: Here, grooming behaviors are visualized in low-dimensional space using t-SNE, which preserves local distances between nearby points. Color indicates behavior type. Behaviors are well-separable by type. B: Example classifications of ethograms of grooming behaviors are shown. Human-algorithm agreement is about 75% (in terms of number of matching frames). (TIF) [file pcbi.1007105.s001.tif]

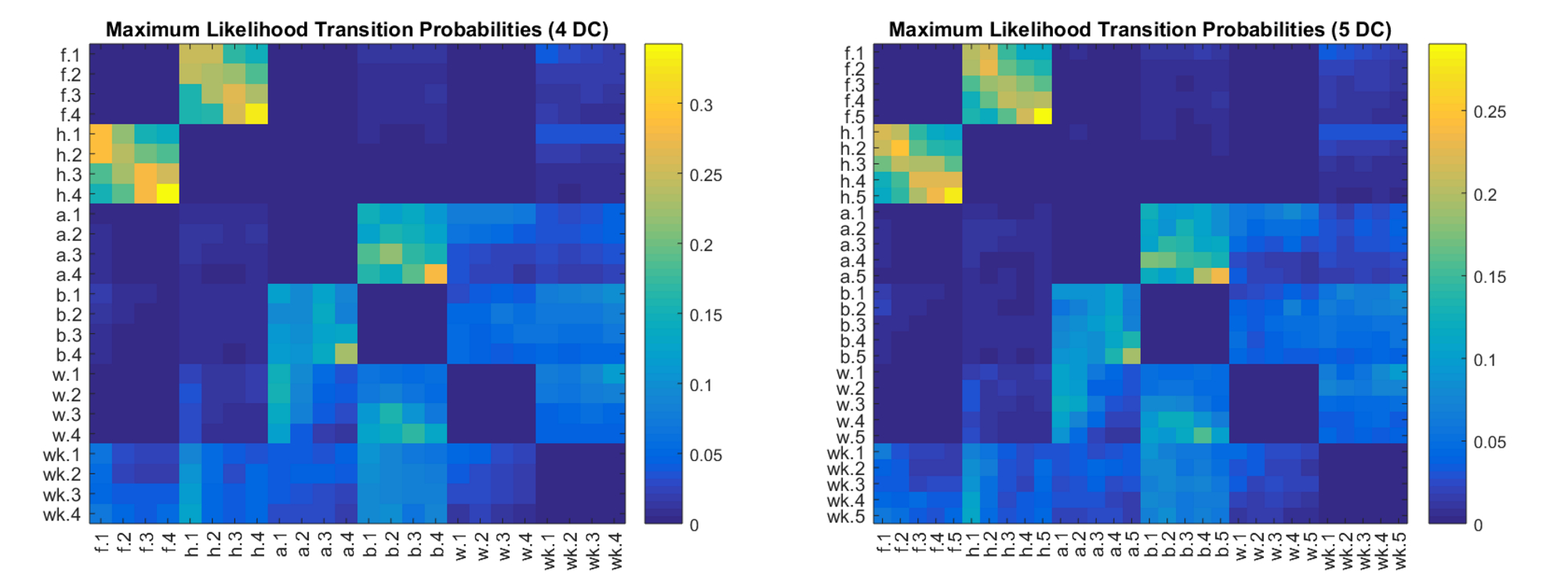

Supplement: S2 Fig — When transition probabilities are calculated from ethograms with 4 (left) or 5 (right) duration categories, fine-grain temporal structure is still present (compare the matrices shown here to those shown in Fig 7). The existence of this trend across binning schemes ensures that duration dependence is a phenomenon present in the data and not an artifact of data processing. (TIF) [file pcbi.1007105.s002.tif]

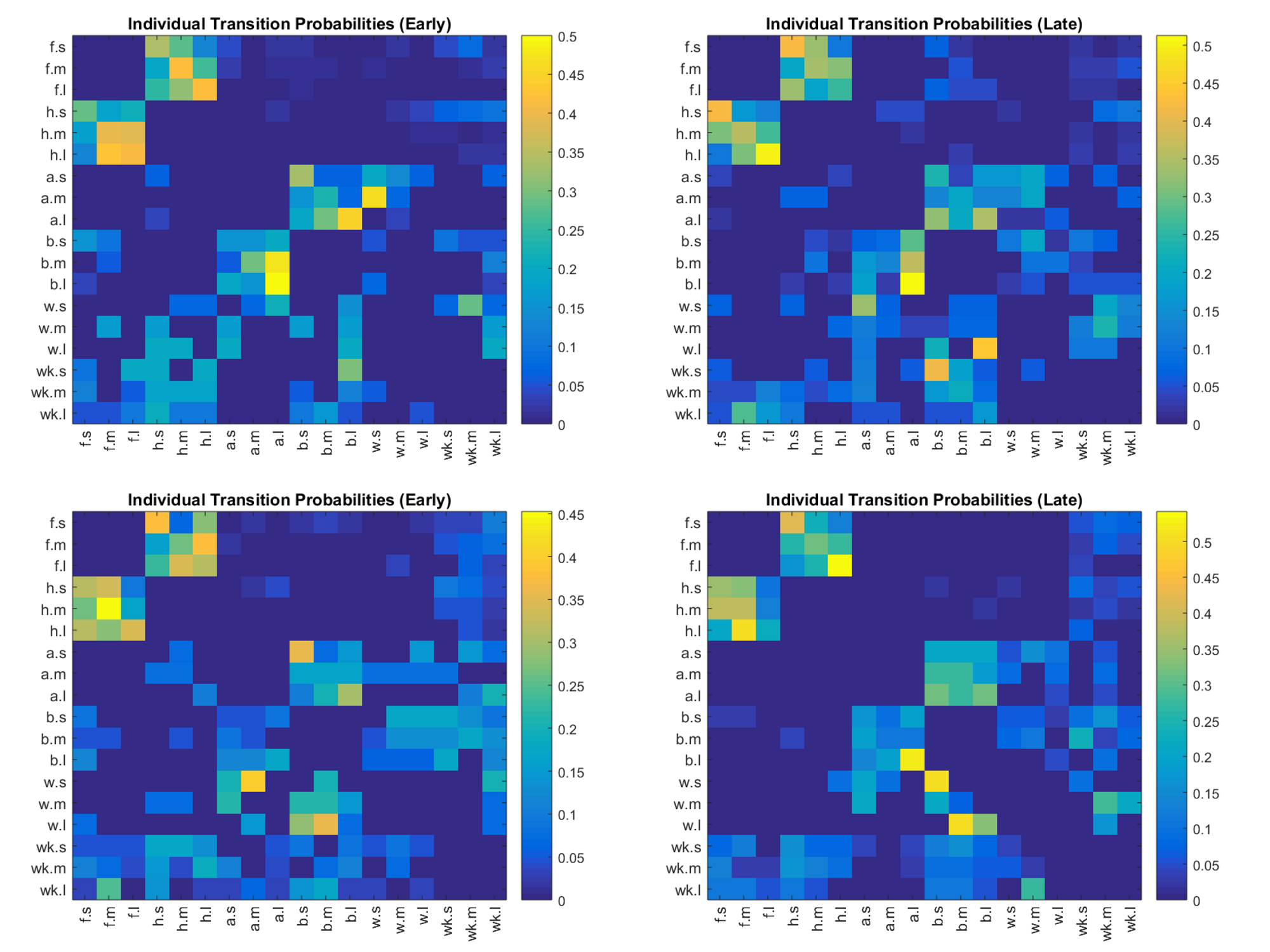

Supplement: S3 Fig — Two examples of individual fly transition probabilities illustrate that, although syntax varies between individuals and over time, individuals are relatively stable and resemble the group average (compare to Fig 9). Though we did not fully characterize individual differences here, future studies will aim to examine individuals more closely. (TIF) [file pcbi.1007105.s003.tif]

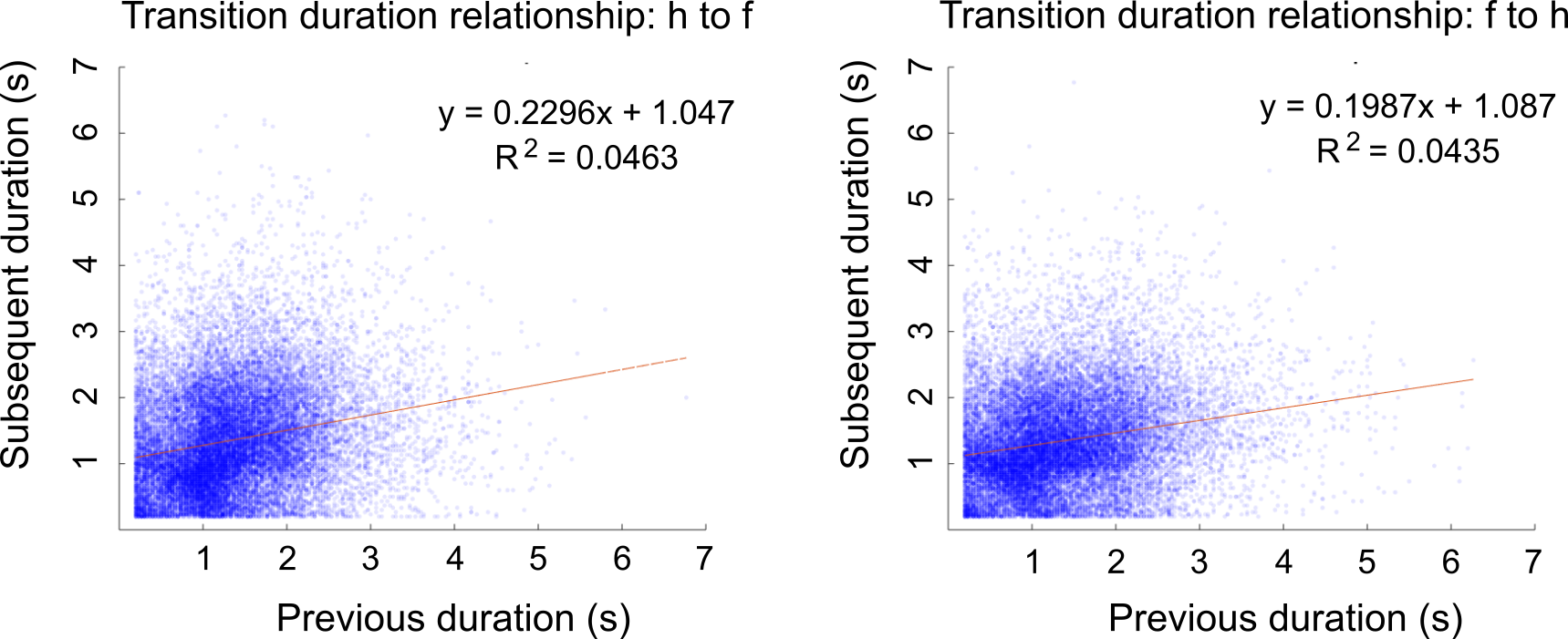

Supplement: S4 Fig — Plotted here is the relationship between consecutive anterior grooming action durations. Linear regression produces a poor fit, indicating that duration dependence is not linear. (TIF) [file pcbi.1007105.s004.tif]

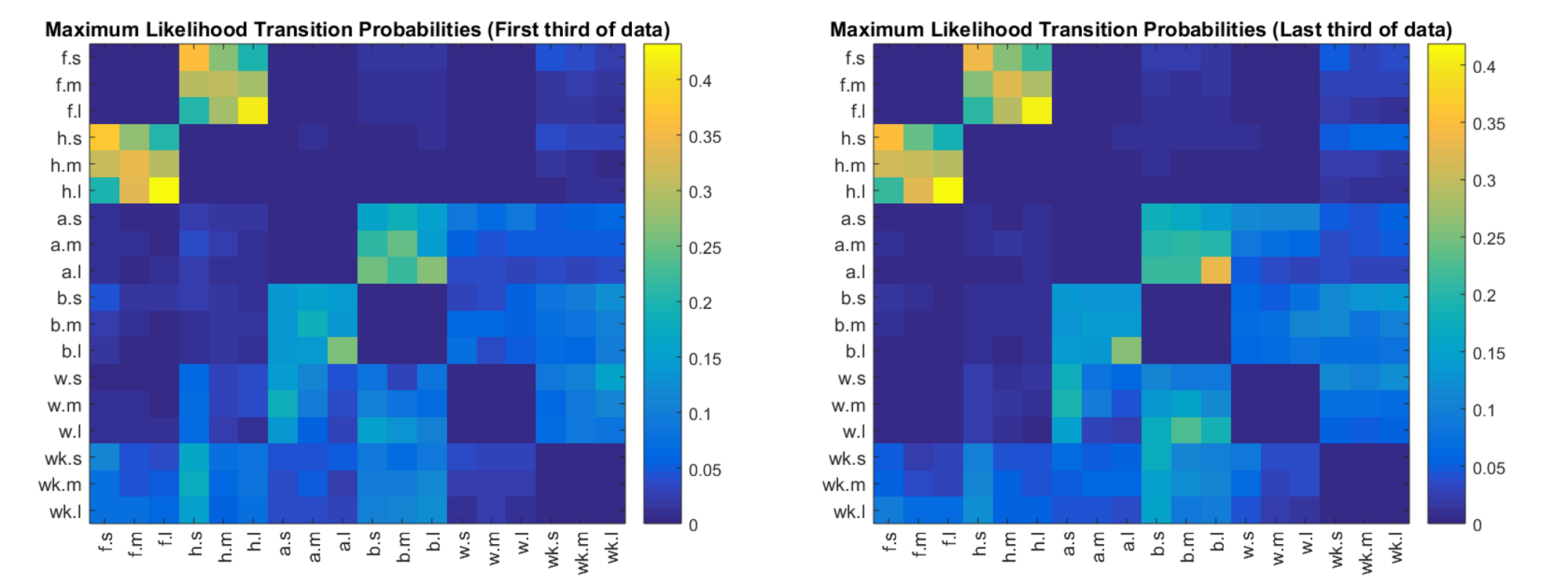

Supplement: S5 Fig — To ensure that our choice of boundary between early and late phases does not alter our results, we partition our data in a stricter manner so that the transition period which occurs during the middle of the grooming progression is excluded. Partitioning the data into thirds produces transition matrices with structure and stationarity similar to those shown in Fig 8. (TIF) [file pcbi.1007105.s005.tif]

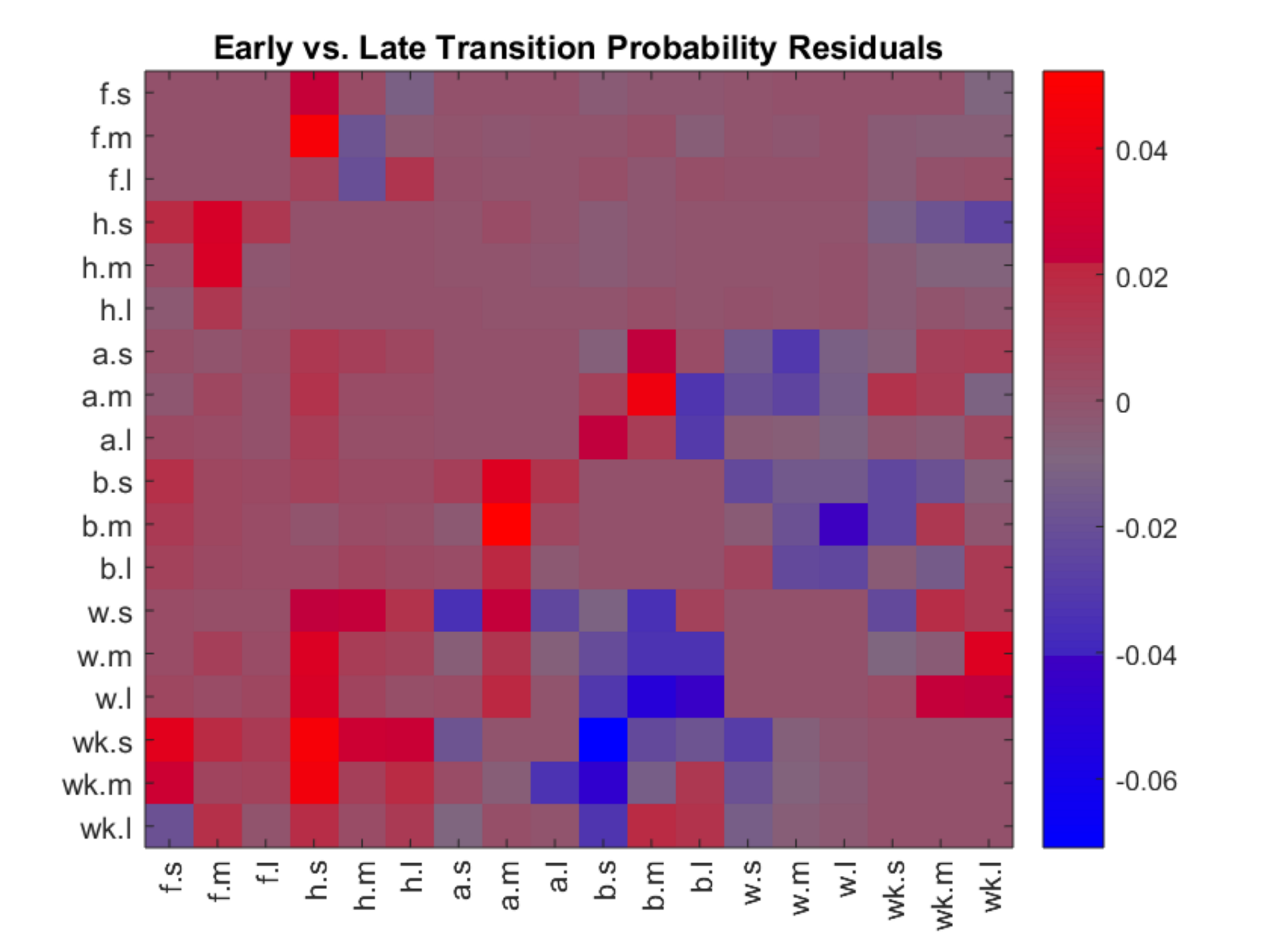

Supplement: S6 Fig — We find that 253 of the 266 (95.1%) non-zero entries change by less than 5% and 168 (63.2%) entries change by less than 1%. This was a very surprising finding and suggests that, although some non-stationarity does exist, it is much smaller than we had expected given that the average time spent performing different behaviors varies between the early and late phases. (TIF) [file pcbi.1007105.s006.tif]

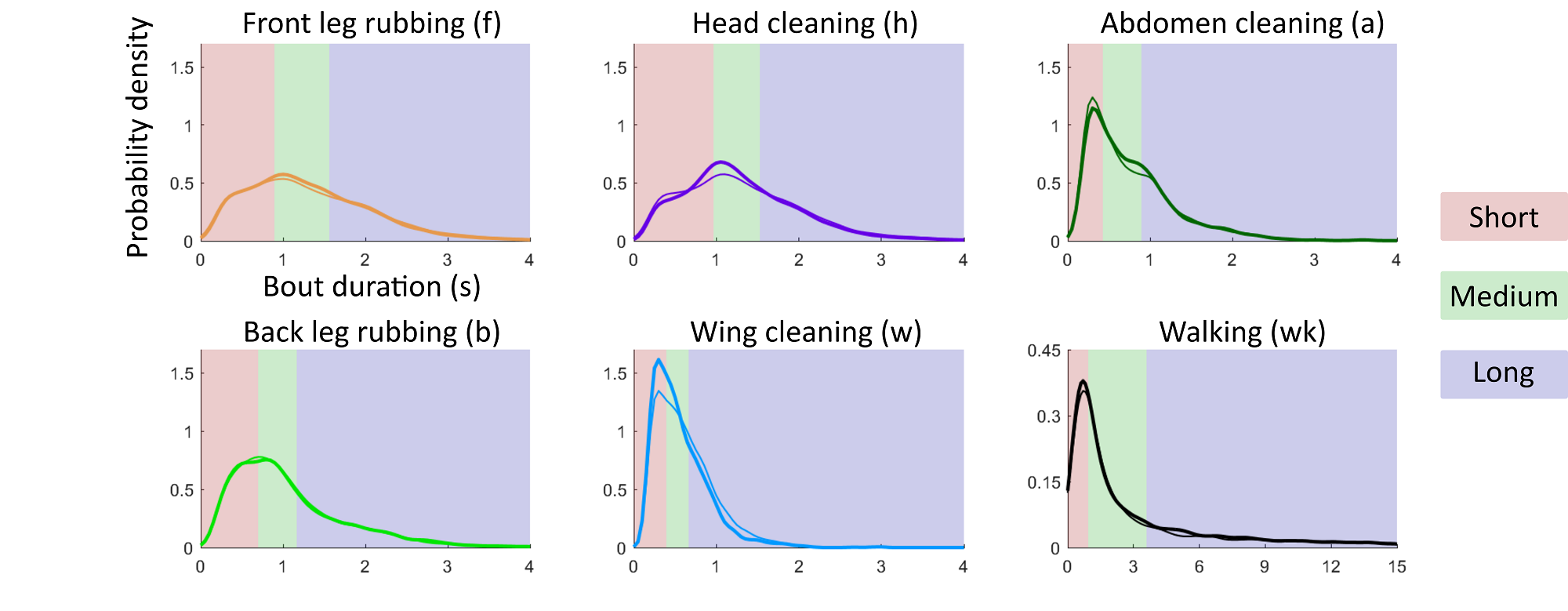

Supplement: S7 Fig — Probability density plots of bout duration distributions illustrate that bout durations remain consistent between early and late phases of grooming. Early phase data is plotted with a thick line. Late phase data is plotted with a thin line. (TIF) [file pcbi.1007105.s007.tif]

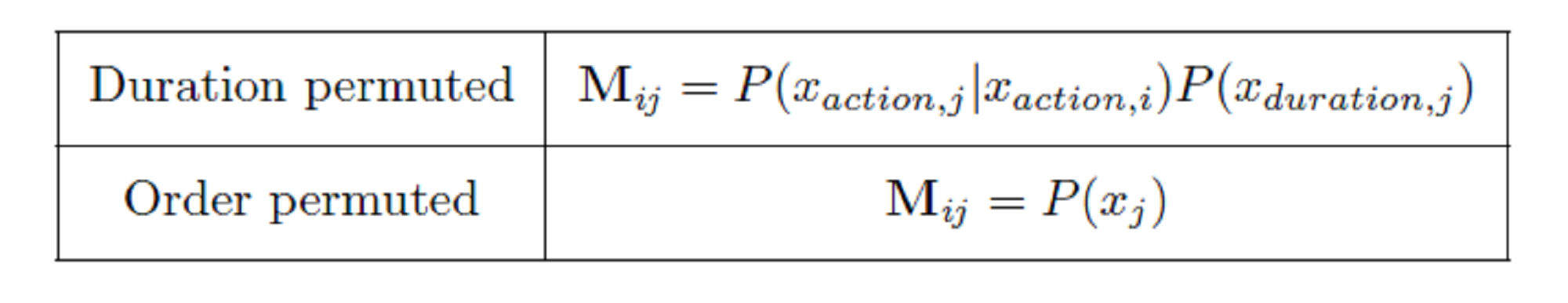

Supplement: S1 Table — Given in S1 Table are the analytic formulas for transition matrix entries, Mij, for each null hypothesis transition matrix. Here, i denotes the row and j denotes the column of M. Note that, in the three duration category case, each state has both an action and duration identifier (e.g. row h.s. corresponds to a head cleaning action in the short duration category). P(xaction,j|xaction,i) is the absolute probability of transitioning from the grooming action represented by row i to the action represented by row j, regardless of duration (this is given by the discrete time transition probability matrix in Fig 7A). P(xduration,j) is the absolute probability of the grooming action represented by row j belonging to the duration category represented by row j. P(xj) is the absolute probability of state j. (TIF) [file pcbi.1007105.s008.tif]
